# miRNA expression patterns in blood leukocytes and milk somatic cells of goats infected with small ruminant lentivirus (SRLV)

**DOI:** 10.1038/s41598-022-17276-y

**Published:** 2022-08-02

**Authors:** Daria M. Urbańska, Justyna Jarczak, Michał Czopowicz, Jarosław Kaba, Karina Horbańczuk, Emilia Bagnicka

**Affiliations:** 1 Department of Biotechnology and Nutrigenomics, Institute of Genetics and Animal Biotechnology, Polish Academy of Sciences, ul Postępu 36A St., 05-552 Jastrzębiec, Poland; 2 Laboratory of Molecular Basis of Behavior, Nencki Institute of Experimental Biology, PAS, Ludwika Pasteura 3, 02-093 Warsaw, Poland; 3 Biobank Lab, Department of Molecular Biophysics, University of Łódź, ul. Pomorska 139, 90-235 Łódź, Poland; 4 Division of Veterinary Epidemiology and Economics, Institute of Veterinary Medicine, Warsaw University of Life Sciences, ul. Nowoursynowska 159c, 02-776 Warsaw, Poland

**Keywords:** Epigenomics, miRNAs

## Abstract

The study aims to determine the selected miRNAs expression in milk somatic cells (MSC) and blood leukocytes (BL) of SRLV-seronegative (SRLV-SN) and SRLV-seropositive (SRLV-SP) goats. A functional in silico analysis of their target genes was also conducted. MiR-93-5p and miR-30e-5p were expressed only in BL, while miR-144 was expressed only in MSC, regardless of SRLV infection. In the SRLV-SP goats, higher miR-214-3p and miR-221-5p levels were found in the MSC than in the BL. Only miR-30e-5p was influenced by the lactation stage in BL in both groups, while only miR-93-5p was altered in BL of SRLV-SN goats. The target gene protein products exhibited contradictory functions, protecting the host from virus on the one hand and assisting viruses in their life cycle on the other. The differential expression of the miRNAs observed between the MSC and BL of SRLV-SP goats may suggest that the local immune response to the infection in the udder differs from the systemic response, and acts independently. Some miRNAs demonstrated different expression between lactation stages. It may be influenced by the metabolic burden occurring in early lactation and its peak. Some of the studied miRNAs may influence viral infection by regulating the expression of their target genes.

## Introduction

Small ruminant lentivirus (SRLV) causes caprine arthritis-encephalitis (CAE) in goats and visna-maedi (VM) disease in sheep. Belonging to the Retrovirus family, and hence occupying the same group as simian immunodeficiency virus (SIV), feline immunodeficiency virus (FIV) and human immunodeficiency virus (HIV)^1^. SRLV itself is not regarded as an immunodeficiency virus^2,3^. SRLV is capable of spreading imperceptibly through herds because it has a long incubation period, and clinical symptoms occur a long time after infection^4^. Thus, the control of transmission is very difficult^5,6^. CAE is caused by a systemic infection, with the main targets of the virus being monocytes, macrophages and dendritic cells, but not lymphocytes. Infection affects the mammary gland and respiratory and musculoskeletal systems in adult goats and the central nervous system in kids, though clinical symptoms are extremely rare in offspring^1,7^.

Our previous findings suggest that SRLV can evade the immune system, or that it activates the immunity only to a slight extent. Differences have been found between SRLV-seronegative (SRLV-SN) and SRLV-seropositive (SRLV-SP) goats regarding the some cytokines or acute phase proteins (APPs) concentrations, with SRLV-SP goats demonstrating lower concentrations of interleukin 1α, (IL-1α) and interleukin 1β (IL-1β) and a higher concentration of serum amyloid A (SAA) in blood serum. In addition, higher concentrations of Il-1α, interleukin 6 (Il-6), and interferon β (IFN-β), and lower concentrations of SAA and ceruloplasmin (Cp) have been observed in milk^8,9^. Elevated concentrations of SAA and haptoglobin (Hp) in the blood serum of goats with clinical CAE compared to healthy or asymptomatic infected goats were also found^10^. It should be stressed that SAA may foster virus multiplication. Jarczak et al.^8^ and Reczyńska et al.^9^ reported that the studied genes demonstrated a slightly different pattern of expression at the transcription level. Some differences at the mRNA level were found in the expression of the *Il-1α, Il-1β, Il-6,* interferon γ (*IFN-γ),* tumour necrosis factor α *(TNF-α), SAA,* and *Hp* genes in blood leukocytes (BL) and of the *IFN-β, IFN-γ, TNF-α,* and *Hp* genes in milk somatic cells (MSC). These differences between mRNA and protein expression indicate that some differential post-transcriptional regulation exists between SRLV-SN vs. SRLV-SP goats. However, it is not clear if they are connected with the effect of the virus but for sure they concern post-transcriptional or post-translational modifications in the MSC and BL.

Epigenetic modification studies have recently advanced to a new level of development. Epigenetic mechanisms such as DNA demethylation, RNA methylation, cytidine acetylation in mRNA, and chromatin modifications may soon become the primary focus of researchers. However, microRNAs (miRNAs) activity is still being investigated as a key regulator of target gene expression. The targeted analysis must focus on a specific pathway and explain the details of the selected process. There is still a lack of knowledge about the molecular processes involved in SRLV infection, including the basis of the pathophysiological processes.

MiRNAs are short sections of non-coding, single-stranded RNA around 21 to 23 nucleotides in length^11^. It is estimated that between 30%^12^ and 60%^13^ of human genes may undergo miRNA regulation, and that approximately 3% of genes encode miRNAs^12^. One miRNA type can be complementary to one or more genes, and one mRNA strand may have binding sites for several miRNAs. MiRNA paired to the 3′ untranslated region of the target gene mRNA can induce mRNA deadenylation, degrade the mRNA, or inhibit the translation process without mRNA degradation. In humans, miRNAs very rarely degrade or cleave mRNA; they are more likely to inhibit the translation process^12–14^. MiRNAs are known to influence a range of biological processes, such as cell proliferation, differentiation, growth, development, and apoptosis^13,15^.

In mammals, many non-coding RNAs participate in the immune response to viral infections and have been found to control virus multiplication. The effect of viral infection of the human respiratory system have been examined^16^, while lungs are one of the main target organs of SRLV in sheep and goats^1,3^. For example, in an in vitro study on A549 human lung epithelial cells (ATCC) and human epithelial type 2 (HEp-2), **miR-24** was found to act against human respiratory syncytial virus (RSV) and influenza virus (IAV)^16^. Moreover, IAV infection was found to be associated with increased **miR-29** expression, which is known to activate the host antiviral response by modulating the pro-inflammatory signalling network^17^, but with reduced **miR-30** expression, which also influences cell apoptosis and proliferation. **MiR-30** also regulates the host cellular response to human metapneumovirus infection^13^. In turn, RSV silences **miR-221** expression, thus inhibiting the apoptosis of infected cells and increasing viral replication and infectivity^18^. Increased expression of **miR-214** is observed in bronchoalveolar stem cells during severe acute respiratory syndrome-associated coronavirus (SARS-CoV) infection; this is believed to stimulate the production of E1A protein and inhibit virus replication^13,19,20^. **MiR-214** is also thought to help the SARS virus particles evade removal by the immune system by inhibiting its replication until the virus transmission is successful^19^. In addition, **miR-24** and **miR-93** have been found to inhibit the replication of vesicular stomatitis virus (VSV) in mice by targeting viral genes^21^. Elevated levels of **miR-24 and miR-30a-5p** were found in the blood serum of patients with rheumatoid arthritis (RA)^22^. It should be stressed that, although the diseases have different aetiologies, CAE is considered the animal model of this disorder^23^. Moreover, **miR-214**, which plays contradictory roles in SARS-CoV infection in humans, also downregulates the expression of the lactoferrin gene in human mammary epithelial cells^24^. Elevated **miR-141** expression has been observed during enterovirus infection of rhabdomyosarcoma cells^25^. Summing up, elevated miRNA expression does not always work in favour of the host immune system: it can also facilitate the replication or spread of pathogens in the host organism.

In the studies of caprine and ovine biology, the subject of research in relation to several forms of RNA e.g.: miRNAs, circle RNAs and lncRNAs (long noncoding RNAs) are mainly those about animals pregnancy^26–29^, functioning of the tissues and organs^30–33^, mammary gland health state^34,35^, or the function of the milk extracellular vesicles^36^. However, currently, only very limited information exists on the participation of miRNAs in SRLV infection. In the most recent study^37^, the analysis of miRNA expression in the lung of sheep infected with VM virus was presented. It revealed several miRNAs that were differentially expressed between VM seropositive groups and uninfected groups. Results describing the expression of miRNAs in the mammary gland and blood of goats with SRLV infection are still not available. Therefore, the present study is restricted to selected miRNAs known to take part in the host response to other viral infections in mammals and for which the sequences for goats were known. The findings are supplemented by an in silico analysis to identify their target genes, also currently known to be involved in the response to viral infections. Furthermore, following on from our previous studies, the present work contrasts the systemic immune response to SRLV infection and the response demonstrated in the udder based on miRNA expression profile.

The aim of this study was to determine the expression of seven miRNAs (chi-mir-214-3p, chi-mir-221-5p, chi-mir-24-5p, chi-mir-29b-3p, chi-mir-93-5p, chi-mir-141-3p, chi-mir-30e-5p) in the BL and MSC of goats infected with SRLV. We also performed an in silico analysis of their probable influence on the genes of the immune system. As no information regarding the miRNA target genes is currently available for goats or other related species in TarBase^38^, the present analysis is based on information taken from human databases.

## Methods

### Animals

The study was conducted on the same animal material as described by Reczyńska et al.^9^. It included 12 Polish White Improved (PWI) and 12 Polish Fawn Improved (PFI) dairy goats. The animals were kept in a loose barn under constant veterinary supervision. Moreover, the animals were examined by two specialists who were Diplomate of the European College of Small Ruminant Health Managements (co-authors JK and MC); no symptoms of clinical mastitis were stated. The goats were fed according to a system developed by the Institut National de la Recherche Agronomique (INRA) of France and adopted by the Research Institute of Animal Production, Poland^39^. Their basic diet consisted of maize silage, wilted grass silage, and concentrates.

Goats in this herd have been routinely serologically tested for SRLV for more than twenty years (each December and June) using commercial ELISA (enzyme-linked immunosorbent assay)^40^, as part of the SRLV eradication program initiated in 1997 following the discovery of seropositive goats. The presence of the virus in the herd was confirmed by its isolation^41,42^. All kids were isolated from their mothers, irrespective of the maternal serological status, and fed colostrum followed by cow milk or milk replacement (Sprayfo Primo Goat Kid, Trouw Nutrition, Grodzisk Mazowiecki, Poland) depending on the year of rearing. Confirmation of infection was based on at least two consecutive positive serological tests (ID Screen MVV/CAEV Indirect-screening test, IDvet, Grabels, France) conducted at six-month intervals. The tests were also performed twice a year during the study to identify new potential infections and to eliminate infected animals from the control group; however, the goats in the SRLV-SN group had registered no positive tests during their entire lives. Irrespective of their serological status, all goats enrolled in the study were asymptomatic probably because of the low virus load: RT-qPCR analysis, performed according to Brinkhof et al.^43^ found the number of the virus copies to be below detection level (data not shown).

The goats were divided into two groups: SRLV-SN (N = 12) and SRLV-SP (naturally infected with SRLV; N = 12). Both groups were identical in terms of breed (6 PWI and 6 PFI) and parity: all were second parity, i.e. young goats, but not primiparous, or more than second parity, i.e. animals who had finished their somatic growth. None of the studied goats were euthanized because of the study and remained in the herd for further commercial use.

All goats were kept under the same environmental conditions, but the groups of SRLV-SN and SRLV-SP goats were separated to avoid possible cross-infection. All were machine milked twice a day, with the SRLV-SN goats being milked first to eliminate the risk of SRLV transfer via milking equipment.

### Sample collection

The milk and blood samples were collected five times during lactation: just after kidding, and on day 30 (early lactation), day 60 (peak of lactation), day 140 (mid-lactation), and day 200 (late lactation) of lactation.

Just before the morning milking, a small amount of foremilk was also collected by hand in a sterile manner. To identify the bacterial pathogens, Columbia agar supplemented with 5% sheep blood and MacConkey agar (bioMérieux, France) were used. Both media were inoculated with 100 μL of milk, and the plates were incubated at 37 °C for 48 h. The bacterial species was identified using VITEK 2 equipment (bioMérieux, France).

One litre of udder milk from each goat was collected in a plastic RNase-free bottle during morning machine milking and centrifuged to obtain a pellet from MSC for RNA isolation. However, before that, 20 ml of representative milk samples from the whole udder milk was collected to the tube with preservative (Microtabs, Bentley, Chaska, Minnesota, USA) to establish the somatic cell count (SCC) in milk using IBCm device (Bentley, Chaska, Minnesota, USA).

The whole blood samples were collected by a veterinarian in 9-ml EDTA tubes for RNA isolation one day after milk sampling.

### Milk somatic cell (MSC) isolation

Just after sampling, one litre of milk was centrifuged at 1500 rpm for 30 min, and the lipid layer and skim milk were discarded. The MSC pellet was transferred to 50-mL Falcon™ Conical Centrifuge Tubes (Fisher Scientific, Warsaw, Poland) and washed with phosphate buffered saline (PBS). Next, the tubes were centrifuged at 1,100 rpm for 15 min; the procedure was repeated twice. The obtained MSC were suspended in 1 ml of TRIzol reagent (Invitrogen, USA) and stored at -80 °C for further analysis.

### RNA isolation from milk somatic cells and blood leukocytes

The MSC pellet, obtained from 1 L of milk centrifugation and washed 2-times in PBS, was homogenized in a tube with silica beads using a FastPrep®-24 Instrument (MP Biomedicals, USA).

Total RNA from MSC and whole blood was isolated using an NucleoSpin miRNA Kit (Macherey–Nagel, Düren, Germany), enabling the isolation of Small and Large RNAs, according to the manufacturer’s protocol.

Qualitative and quantitative analyses of RNA were performed using a NanoDrop 2000 spectrophotometer (Thermo Fisher Scientific, Wilmington, DE, USA) and the Bioanalyser 2100 (Agilent, Santa Clara, USA) according to the attached protocols. Only RNA samples with ratio A260nm/280 nm between 1.9 and 2.2 and RIN ≥ 7.0 were selected for further analysis. The RNA samples were stored at − 80 °C.

### miRNA gene expression

Seven miRNAs (chi-mir-214-3p, chi-mir-221-5p, chi-mir-24-5p, chi-mir-29b-3p, chi-mir-93-5p, chi-mir-141-3p, and chi-mir-30e-5p) were selected based on their known involvement in viral diseases; in addition, of the mi-RNAs given above, only these seven sequences were available in miRBase at that time^44^, and could serve as templates for primers. The expressions of genes was measured according to the manufacturer’s instructions using EPIK™ miRNA Select Assays (Bioline, Germany). The total 100 ng RNA from each samples were converted into cDNA using the EPIK™ miRNA RT kit (Bioline, Germany). The cDNA was diluted 1:10. Total volume of sample was 20 µl and was contained 4 µl of cDNA, 10 µl PCR Master Mix and 2 µl primer. The sequences of mature miRNAs used in the analysis are shown in Table 1. Real-time PCR was performed using the LightCycler 480 System (Roche, Switzerland). U6, small nuclear RNA (CAAGGATGACACGCAAATTCG), was used as a reference^32^.

**Table 1:** Sequences of mature miRNA and miRBase accession number.

miRNA name	Accession number in miRBase^a^	Sequences of mature miRNA
chi-mir-214-3p	MIMAT0036059	UACAGCAGGCACAGACAGGC
chi-mir-221-5p	MIMAT0036068	ACCUGGCAUACAAUGUAGAUU
chi-mir-24-5p	MIMAT0036090	GUGCCUACUGAGCUGAUAUC
chi-mir-29b-3p	MIMAT0036115	UAGCACCAUUUGAAAUCAGU
chi-mir-93-5p	MIMAT0036305	CAAAGUGCUGUUCGUGCAGGUAG
chi-mir-141-3p	MIMAT0035962	UAACACUGUCUGGUAAAGAUGG
chi-mir-30e-5p	MIMAT0036128	UGUAAACAUCCUUGACUGGAAGCU

^a^miRbase^44^.

### Bioinformatic analysis

The Database for Annotation, Visualization and Integrated Discovery (DAVID) was used to select the genes whose functions are limited to immunity-related, antiviral response or viral processes^45^. Following this, a functional in silico analysis for human miRNA genes was conducted based on the DIANA-tools-TarBase v8.0, (The Database for Annotation, Visualization and Integrated Discovery (DAVID) v6.8)^38^, UniProtKB/Swiss-Prot UniProt release 2020_03 Apr-22, 2020^46^ and GeneCards databases^47^ (term of the accession—May–June 2020 and April 2021). The target genes were selected on the basis of a prediction score higher than 0.80 based on information available in TarBase^38^. In addition, miRNet software^48^ (term of the accession—April 2021) was used to visualize the networks between studied miRNA and their target genes and the networks between miRNAs and diseases. Using miRNet software and then KEGG pathway module, functional analysis was performed for all target genes for all miRNAs expressed in MSC and BL, separately.

### Statistical analysis

To identify differences in miRNA expression between the groups of animals and type of biological material, analyses of variance was performed using the general linear model (GLM) with Tukey–Kramer tests (SAS/STAT, ver. 9.4; 2002–2012). The model was as follows:

$$y_{ijklm} = \mu + a_{i} + SRLV_{j} + TM_{k} + \left( {SRLV*TM} \right)_{jk} + \left( {SRLV*SL} \right)_{jl} + e_{ijklm}$$

where y_ijklm_ trait value (gene expressions); μ overall mean; a_i_ random effect of the animal (i = 1,…,24); SRLV_j_ fixed effect of the small ruminant lentivirus infection/lack of infection (j = 1, 2); TM_k_ fixed effect of the type of biological material (k = 1,2); SL_l_ fixed effect of the stage of lactation (l = 1,…,5); (SRLV * TM)_jk_ fixed effect of the interaction of the health state of the animals (seropositive or seronegative) and the type of biological material (blood or milk) (kj = 1, … 4); (SRLV * SL)_jl_ fixed effect of the interaction of the health state of the animals (seropositive or seronegative) and the stage of lactation (jl = 1, … 10); e_ijklm_ random error.

The normality of the distribution of all traits was checked using the univariate procedure (SAS/STAT, ver. 9.4; 2002–2012), and values for the levels of the miRNAs were subjected to natural logarithm transformation. The final model did not contain the year of sampling, breed, number of lactation, lack/presence of bacteria, age of infection, or time since infection, because their impact was not reported in our preliminary statistical analysis.

### Ethical approval

The study was carried out in compliance with the ARRIVE guidelines. All applicable international, national and/or institutional guidelines for the Care and Use of Animals were followed. The experimental protocols were approved by the the 3rd Local Ethics Committee for Animal Experimentation in Warsaw; (permission No. 31/2013), following all relevant guidelines and regulations. All goats belonged to the Experimental Farm at the Institute of Genetics and Animal Breeding in Jastrzębiec, near Warsaw, Poland. The Experimental Farm is an integral part of the Institute and maintains a flock of 60 dairy goats of the Polish White Improved, and Polish Fawn Improved. The animals remained under the constant care of a veterinarian (employee of the Institute). Furthermore, the study did not employ anesthesia and euthanasia methods. The owner of the herd gave us a written permission regarding the use of the goats in this study. This article does not contain any experiments on human subjects performed by any of the co-authors.

## Results

### Comparison of miRNA transcript levels between the MSC and BL of SRLV-seropositive vs. SRLV-seronegative goats

Table S1 shows the average SCC (SD) in goat milk collected at each sampling time for the study. The SCC in all points of sampling was low and range between 313 × 10^3^ (SD = 256 × 10^3^) at the beginning of lactation till 620 × 10^3^ (SD = 606 × 10^3^) at the end of lactation. All studied milk samples were free from bacterial pathogens. While environmental bacteria were present at similar levels in the milk of both groups of animals included in that study, they did not influence miRNA gene expression (data not shown). Low SCC and lack of environmental bacterial influence on miRNA expression both imply that animals had no subclinical mastitis.

**Figure 1:**
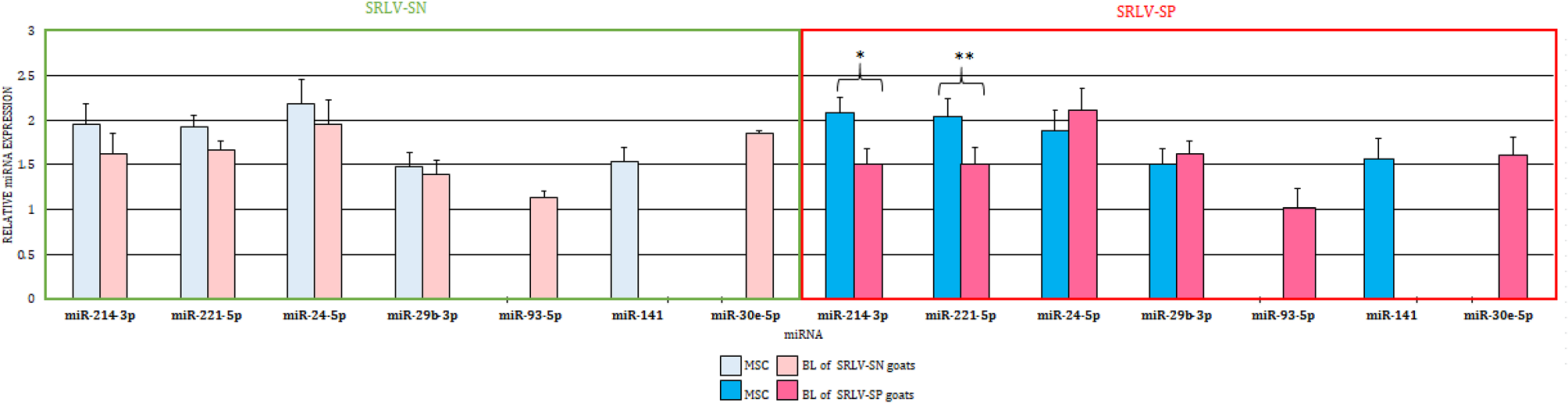
Differences (LSMEANS, ± SE) between miRNA levels in milk cells and blood leukocytes of SRLV-seronegative and SRLV-seropositive goats. The value within the same miRNA with star(s) differs significantly between milk somatic cells (MSC) and blood leukocytes (BL): * at *p* ≤ 0.05 and ** at 0.05 < *p* ≤ 0.01. *SRLV-SN* small ruminant lentivirus seronegative goats, *SRLV-SP* small ruminant lentivirus seropositive goats.

The first part of the present study compared the differences in miRNA levels between the BL and MSC of both SRLV-SN or SRLV-SP goats in separate analyses. In both groups of material, as well as in both groups of goats, four of the seven analyzed miRNAs were found: miR-214-3p, miR221-5p, miR-24-5p, and miR-29b-5p. However, in both the SRLV-SN and SRLV-SP goats, miR-141-3p transcripts were found only in MSC, while miR-93-5p and miR-30e-5p were found only in BL; these miRNAs were found at similar levels in both groups of animals, regardless of SRLV infection (Fig. 1). In the SRLV-SN goats, four miRNAs did not demonstrate any differences in expression between the MSC and BL; however, in the SRLV-SP goats, miR-214-3p (*p* ≤ 0.05) and miR-221-5p (0.05 < *p* < 0.1) were lower in the BL than in the MSC.

### Comparison of miRNA transcript levels in MSC or BL between SRLV-seropositive and SRLV-seronegative goats

**Figure 2:**
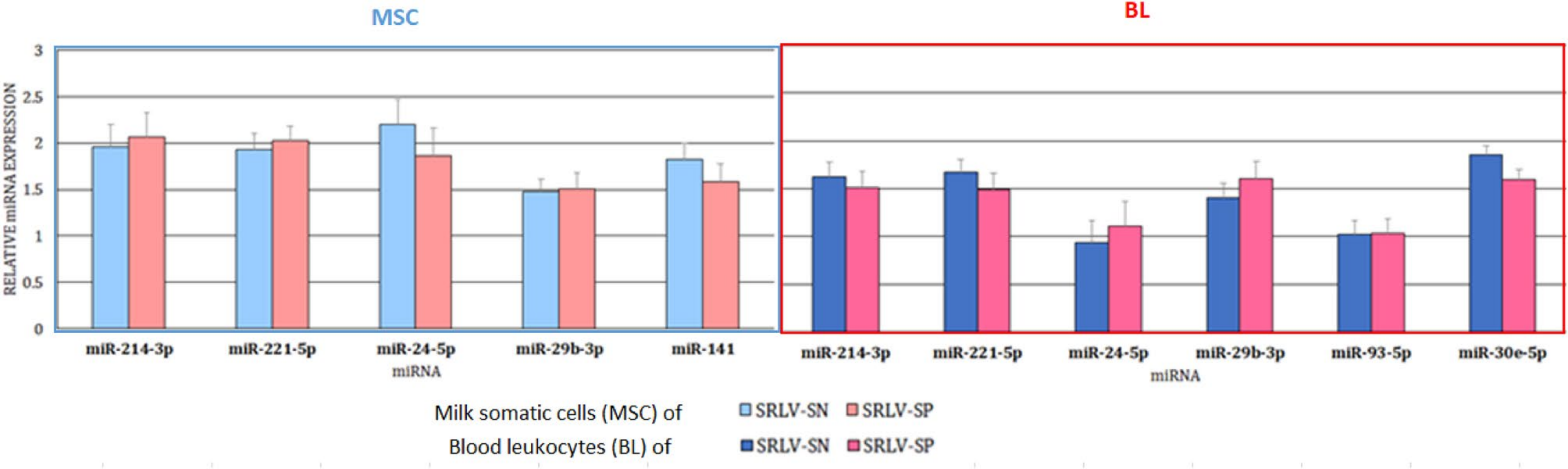
Comparison of the miRNA levels (LSMEANS, ± SE) between SRLV-seronegative and SRLV-seropositive goats in their milk somatic cells or blood leukocytes. *SRLV-SN* small ruminant lentivirus seronegative goats, *SRLV-SP* small ruminant lentivirus seropositive goats.

The second part of the study compared the miRNA expression patterns between SRLV-SP and SRLV-SN goats in their BL or MSC, separately. No differences in the expressions of miR-214-3p, miR-221-5p, miR-24-5p, or miR-29b-3p were found between SRLV-SP and SRLV-SN goats in either MSC or BL (Fig. 2).

However, as was previously mentioned, in both the SRLV-SN and SRLV-SP goats, miR-141-3p expression was noted in the MSC and not in the BL; conversely, miR-93-5p and miR-30e-5p expression was observed only in the BL, and not the MSC (Fig. 1). Nevertheless, the expression of each miRNA, in both MSC and BL, was similar between both SRLV-SN and SRLV-SP.

### Functional analysis of studied miRNAs

A functional analysis of the studied miRNAs and their target genes involved in viral infections, based on human databases, is shown in Table S2 (supplementary files). Human databases were selected due to the paucity of information available in the goat and bovine databases. All biological processes involving the studied miRNAs, or rather the protein products of their target genes (Table S2), are listed and described in Table S3. MiR-24-5p, and miR-141-3p are not included due to the lack of information available on their target genes in TarBase v.8^38^. The full lists of the target genes for miR-29b-3p, miR-30e-5p, miR-93-5p, mir-214-3p, and mir-221-5p are given in Tables S4, S5, S6, S7, and S8 (supplementary files) respectively. Moreover, the relationship between these miRNAs and their target genes identified using miRNet software are visualised in Fig. S1 for MSC, and in Figs. S2 and S3 for BL. Three miRNAs, namely miR-29b-3p, miR-141-3p and mir-214-3p, expressed in MSC, have two common genes: hepatoma-derived growth factor (*HDGF*) and phosphatidylinositol 3,4,5-trisphosphate 3-phosphatase and dual-specificity protein phosphatase PTEN (*PTEN*). In contrast, miR-221-5p has only several common genes with miR-141-3p, and mir-214-3p, but not with miR-29b-3p (Fig. S1). In BL, nuclear receptor coactivator 3 (*NCOA3*) was found as a common gene for four miRNAs: miR-29b-3p, miR-93-5p, and mir-214-3p, mir-30e-5p (Figs. S2 and S3).

Despite the lack of information of the target genes for mir-141-3p in TarBase^38^, miRNet identified 145 targets, including insulin-like growth factor 1 receptor (*IGFR1*), stearoyl-CoA desaturase 5 (*SCD5*), peroxisome proliferator-activated receptor alpha (*PPARA*), two isoforms of CDC25 genes, viz*.* M-phase inducer phosphatase 1 (*CDC25A*) and M-phase inducer phosphatase 3 (*CDC25C*), as well as genes from STAT or MAPK families (Fig. S4). According to miRNet, this miRNA is involved in hepatitis B virus (HBV) infection (Fig. S5).

The Gene Ontology (GO) details regarding the Biological Processes, Cellular Components and Molecular Functions of the target genes for all the above-mentioned miRNAs are shown in Tables S9, S10, S11, S12, S13, and S14a–c while the results of the KEGG pathway analysis for target genes of all miRNAs expressed in MSC and BL are gathered in Tables S15 and S16, respectively. While no GO category was directly associated with any processes concerning SRLV infection, several other pathways were identified, e.g. *HTLV-I infection*, *Influenza A*, *Hepatitis C* pathways in both types of biological material, and *Herpes simplex infection* pathway in MSC, while *Epstein-Barr virus infection* pathway in BL.

### miRNA expression pattern during lactation in the MSC or BL of SRLV-seropositive or SRLV-seronegative goats

**Figure 3:**
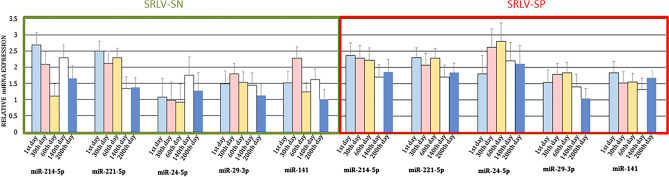
LSMEANS (± SE) of the five miRNA levels in milk somatic cells of SRLV-seronegative and SRLV-seropositive goats during lactation. Stages of lactation: just after kidding, on days 30th (early lactation), 60th (peak of lactation), 140th (mid-lactation), and 200th (late lactation) of lactation.

The third part of the study examined the expression of five miRs in the MSC of SRLV-SN and SRLV-SP (Fig. 3) goats over the course of lactation. No differences were found between the stages of lactation.

**Figure 4:**
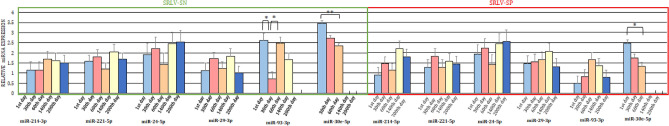
LSMEANS (± SE) of the miRNA levels in the blood leukocytes of SRLV-seronegative and SRLV-seropositive goats during lactation. The values within the same gene with star(s) differ significantly between stages of lactation: ** at *p* ≤ 0.01 and * at *p* ≤ 0.05. Stages of lactation: just after kidding, on days 30th (early lactation), 60th (peak of lactation), 140th (mid-lactation), and 200th (late lactation) of lactation.

The expression profiles of the six miRNAs identified in the BL of SRLV-SN and SRLV-SP goats during lactation are shown in Fig. 4. The expression of miR-93-3p (*p* ≤ 0.05) and miR-30e-5p (*p* ≤ 0.01) in the BL of SRLV-SN goats and miR-30e-5p (*p* ≤ 0.05) in the BL of SRLV-SP goats differed between some stages of lactation. In the SRLV-SN goats, miR-93-3p expression was higher on day 1 of lactation, i.e. immediately after kidding, and on day 60 at the peak of lactation, compared to day 30, i.e. early in lactation; however, no expression was observed on day 200 (late lactation). In contrast, miR-30e-5p expression peaked on day 1 of lactation compared to day 60 in both groups of animals; however, no transcripts were found during mid-lactation or late lactation. A functional analysis of miR-93-3p and miR-30e-5p is presented in Table S2.

## Discussion

### A comparison of miRNA expression with functional analysis between MSC and BL for SRLV-SP or SRLV-SN goats

The differences in immune-related gene expression patterns observed previously between mRNA and protein levels^8,9^ are most likely explained by related to virus post-transcriptional regulation. Gene expression is believed to be regulated by miRNA through degradation or translational silencing of mRNA^14^. Although studies on goat miRNAs are still limited, a number of miRNAs have been identified in other animals that are also responsible for regulating the expression of genes involved in viral infections. For example, a number of miRNAs that are differentially regulated between SRLV-SN and -SP sheep, such as oar-miR-21, oar-miR-148a and oar-let-7f as well as has-miR-28a/b, hsa-miR-146a, hsa-miR148a, hsa-miR-155, and hsa-let-7c, may have potential implications for the host-virus interaction^37^. Unfortunately, this information was published too late to influence the design of the present study, and the goat homologs of those miRNAs were not included, except for chi-miR-29b-3p which was also investigated in cited research.

In the present study, miR-214-3p and miR-221-5p were more abundant in the MSC of the SRLV-SP goats than in the BL. In humans, miR-214-3p appears to target the signal transducer and activator of transcription 3 (*STAT3*)*,* La-related protein 1 (*LARP1*), and protein cornichon homolog 1 (*CN1H1*) genes while miR-221-5 targets heterogeneous nuclear ribonucleoprotein A1 (*HNRNPA1*). These genes may play a role in hepatitis C virus (HCV), human herpesvirus 8 (HHV-8), VACV, influenza A virus (H1HN1), Dengue (DENV) or Zika virus infections (Table S2). The *STAT3* gene expression inhibition due to the elevated level of miRNA-214-3p found in the MSC of SRLV-SP goats might inhibit the infection of further cells by the virus, since the STAT3 exhibits proviral activity (*i.a.* HBV, herpes simplex virus 1 (HSV-1), varicella zoster virus, measles virus, and cytomegalovirus (CMV)); however, it also demonstrates antiviral activity against enterovirus 71, SARS-CoV and human metapneumovirus infection^48^. Nevertheless, this protein is involved in a range of biological processes including viral processes (GO:0016032) (Table S3). This may mean that STAT3 assists the entry of the virus into the cell, its transport to replication sites or its exit from the cell through specific interactions with the virus. STAT3 is also activated by the Nef protein of HIV^48^ as well as in response to various cytokines including INFs and Il-6 (Table S2). Interestingly, elevated concentrations of INF-β and Il-6 have previously been observed in the MSC of SRLV-SP compared to SRLV-SN goats^8^. This may mean that although the expression of cytokines which activate STAT3 may be elevated, the elevated expression of miR-214-3p may inhibit *STAT3* gene expression at the transcription stage. However, pairing a miRNA with the mRNA of the target gene could cause either its degradation or elevate its copy number^14^. Therefore, to identify the processes occurring in MSC infected with SRLV, further studies examining the expression of miRNA-214-3p, STAT3 and a broad panel of cytokines are needed.

LARP1 is the next target gene of miR-214-3p. The protein supports the replication of DENV and increases VACV infection in parallel with CN1H1 and HNRNPA1, which also increases VCAV infection and may play a role in HCV RNA replication; it is also believed to be involved in viral processes (GO:0016032) (Tables S2 and S3). Therefore, their inhibition by miRNA may protect host cells from infection.

To summarise, elevated expression of miR-214-3p and miR-221-5p in the MSC may inhibit the activity of the proteins that directly support invasion by viruses, and it has been suggested that in the udder, it is the immune cells that fight the virus^8,9^. Moreover, Reczyńska et al.^9^ propose that the presence of a higher SAA level in the blood serum of SRLV-SP goats may support viral replication, as SAA demonstrates chemotactic activity toward leukocytes, potentially inhibits the production of antibodies by B lymphocytes and inflammatory reactions, and stimulates the differentiation of monocytes to macrophages, which is essential for viral multiplication. Similarly, Jarczak et al.^8^ report decreased expression of IL-1α and IL-1β proteins in the blood serum of infected goats, suggesting that the virus has the ability to impair their immune system and prevent it from fighting the disease.

Hence, it is difficult to conclude clearly whether overexpression of these two miRNAs in MSC supports or inhibits virus infection. On the one hand, they inhibit certain target genes whose product demonstrates proviral activity, while on the other, they inhibit genes whose products protect the host cells from virus infection.

However, our present results are consistent with previous findings that the local immune response of the mammary gland differs from the systemic immune response^8^. Unfortunately, our in silico analysis did not reveal any APP or cytokine as a target gene of the studied miRNAs; however, STAT3, a protein produced by one of the target genes, is activated by a range of cytokines, including the IL-6 cytokine family. Moreover, the protein product of the elongation factor 1-alpha 1 (*EEF1A1*), a target gene of miR-30e-5p, directly regulates transcription of a number of genes: IFN-gene and sequestosome 1 (*SQSTM1)*, ATP-dependent DNA/RNA helicase DHX36 (*DHX36*) and phosphatidylinositol 3-kinase regulatory subunit alpha (*PIK3R1*). These genes are targets of miR-93-5p, miR-303-5p, and miR-29b-3p, respectively, and are involved in different cytokine signalling pathways.

Therefore, it would be beneficial for future studies to expand the number of analysed miRNAs to include those found to be associated with cytokine or APP expression in other diseases. Recent studies, for example, found miR-146a and miR-155 to regulate the major protein cascades of cytokine signalling pathways and inflammation in human RA: CAE serves as a model for RA^49^. Unfortunately, the experimental part of our study had already been finished when this information was published.

The fact that miR-141-3p was only expressed in MSC and miR-93-5p and miR-30e-5p in BL indicates that their expression in goats is tissue specific. MiR-93-5p, observed only in BL, and miR-24, observed in both BL and MSC, are involved in the direct inhibition of VSV replication by attaching to the regulatory parts of two genes associated with the virus^21^. However, miR-30e-5p influences the activity of a number of human viruses, including HIV-1, HCV, IAV and HHV-1, through its target genes (Table S1). It was found that miR-30e intensifies the innate immune response in cells and reduces HBV load^50^. However, it is unlikely that these miRNAs play a similar role in SRLV because no differences in expression could be seen between the SRLV-SN and SRLV-SP groups.

The in silico analysis (Table S2) identified five target genes involved in the immune defence, for miR-93-5p. Of these, mitogen-activated protein kinase kinase kinase 5 (MAP3K5) and chromobox protein homolog 5 (CBX5) are involved in viral processes (GO:0016032). In addition, the MAP3K5/ASK1 complex supports the host defence against various pathogens, while CBX5 interacts with Human polyomavirus 2 agnoprotein, and hence probably with the agnoprotein of Simian virus 40 (SV40); these processes disrupt CBX5 and lamin-B receptor association, resulting in destabilisation of the host cell nuclear envelope. SQSTM1 interacts with vif, a protein of HIV; serine/threonine-protein kinase N2 (PKN2) stimulates HCV replication by interacting with HCV NS5B protein, and as such is involved in viral RNA genome replication (GO:0039694). Rho-associated protein kinase 2 (ROCK2) inhibits HCV infection while increasing VAVC infection. Therefore, further studies are needed to explain the role of miR-95-5p in SRLV infection.

An increased level of miR-141 has been noted in enterovirus infection; it is believed to accelerate viral translation and facilitate the spread of the virus in the body^25^. Moreover, the miRNet in silico analysis found this miRNA to be involved in HBV infection (Fig. S5). In the present study, similar expression levels of miR-141-3p were observed in the MSC of both SRLV-SN and SRLV-SP goats with no expression in BL, suggesting that this miRNA does not participate in the process of SRLV infection. TarBase in silico analysis did not identify any target gene for miR-141-3p; however, almost 150 target genes were identified using miRNet (Fig. S4), including *IGFR1*, *SCD5*, *PPARA*, isoforms A and C of *CDC25*, as well as signal transducer and activator of transcription 4 and 5 (*STAT4, STAT5*), mitogen-activated protein kinase 9 (*MAPK9*), and *MAPK14*. According to the GenomeRNAi database^51^, the protein products of *IGFR1*, *SCD5*, *PPARA* and *STAT4* increased VACV infection, while those of *SCD5* decreased Sindbis virus (SINV) infection and those of *PPARA* influenza A virus infections. The roles of CDC25 and MAPK14 also appear ambiguous with regard to VACV infection; however, a study of the UniProt database^46^ found them to be involved in viral processes (GO:0016032; Table S2) or cellular response to virus (GO:0098586; Table S2), respectively. In turn, STAT5A was found to resist VACV-A4L infection and decrease its replication^51^. Interestingly, MAPK9 appears to support VACV infection but resist VACV-A4L infection; moreover, it was found to decrease Simian viru s 40 (SV40) infection while increasing Human cytomegalovirus (HCMV) strain AD169 replication.

Three miRNAs (miR-29b-3p, miR-141-3p, mir-214-3p) expressed in MSC, have two common genes: hepatoma-derived growth factor (*HDGF*) and *PTEN*. The HDGF protein plays an ambiguous role in VACV infection^51^; however, Yang et al.^52^ speculate that HDGF could act as a transcriptional repressor. Similarly, the role played by PTEN in virus infection remains unclear. *NCOA3* gene was a target for four miRNAs in BL, viz*.* miR-29-3p, miR-30e-5p, miR-93-5p and miR-214-3p; it has also been found to potentially increase VACV infection but decrease human papilloma virus 16 (HPV16) pseudovirus infection^47^.

As miRNAs may regulate more than 60% of human genes, the expression of the target genes identified by the present in silico analysis, requires further study. The target genes of the overexpressed miRNAs identified herein show ambiguous activity toward viral infections, but it is possible that not all of those target genes are triggered by overexpressed miRNAs. In addition, since miRNA-mRNA pairing influences mRNA translation, as well as the stability of the miRNA^14^, further extensive in vivo studies are needed to fully understand the dependencies between mRNAs and miRNAs.

### A comparison of miRNA expression with functional analysis over the lactation course for MSC or BL and SRLV-SP or SRLV-SN goats

MiR-30e-5p was the only miRNA to be influenced by the stage of lactation in the BL of both SRLV-SN and SRLV-SP goats. Its presence regulates the expression of genes involved in the biology of a number of human viruses, including HIV-1, HCV, influenza viruses and HHV-1 (Table S2). Most of the protein products of these target genes, such as DHX36, DNA damage-inducible transcript 4 protein (DDIT4), Msx2-interacting protein (SPEN), EEF1A1, or ubinuclein-1 (UBN1) act against viruses, being involved in the *defense response to virus* biological processes (GO:0051607). However, while SNARE-associated protein Snapin (SNAPIN), nuclear ubiquitous casein and cyclin-dependent kinase substrate 1 (NUCKS1) and cyclin T2 (CCNT2) positively regulate the processes essential for the viral life cycle, ROCK2, Ras-related protein Rab-7a (RAB7A), E3 SUMO-protein ligase RanBP2 (RANBP2), or SPEN both inhibit and promote viral spread.

Zhu et al.^53^ report the presence of an elevated level of miR-30e* during DENV infection and conclude that this miRNA inhibits virus replication by promoting IFN-β expression; however, in the present study, its expression varied with the stage of lactation but not the presence of SRLV infection. Therefore, only changes in its expression observed in the BL may be associated with the metabolic burden of the body and any disturbances in homeostasis at critical points during lactation (the perinatal period, the lactation peak); these factors do not appear to be associated with any changes in the MSCs. This is supported by the fact that the protein products of some target genes of miR-30e-5p are associated with processes essential for ensuring the fat content in milk, such as the de novo biosynthesis of long-chain saturated fatty acids (fatty acid synthase, FASN), transport of long-chain fatty acids (FABP3), or the synthesis of triglycerides (diacylglycerol O-acyltransferase 2. DGAT2)^47^. The lack of expression of this miRNA in MSC may suggest that its target genes in milk are not inhibited throughout the whole lactation period.

The results obtained in our study are not entirely consistent with those presented by Chen et al.^54^, who note that miR-30e-5p expression peaks during the early lactation and subsequently decreases in Saanen goats’ mammary epithelial cells (GMEC); in contrast, in the present study, miR-30e-5p was not found in MSC, which also includes living exfoliated epithelial cells^55^. Chen et al.^54^ identified miR-30e-5p transcripts in many dairy goat organs (heart, liver, spleen, lungs, kidneys, muscles, stomach tissues, GMEC) with the highest expression in GMEC; miR-30e-5p expression was observed in BL in the present study.

In humans, miR-93-5p regulates the expression of genes involved in processes related to the innate immune response against various pathogens, including several viruses (Table S2); the activities of all known target genes of miR-93-5p are discussed above, as the miRNAs are expressed in BL but not in MSC. In the present study, in the BL of SRLV-SN goats, its expression was lowest during early lactation and absent during late lactation, but highest during the perinatal period and at the lactation peak. Contrary to our findings, a higher level of miR-93 transcripts was previously found during the late lactation stage in GMECs, with a lower level observed at lactation peak^56^. Therefore, further research is needed on the role and functions of this miRNA in both SRLV-SN and SRLV-SP goats at different stages of lactation.

The first steps of our study were conducted by Jarczak et al.^8^ and Reczyńska et al.^9^ on cytokine, APP and cathelicidin genes expression at the mRNA and protein levels; however, our knowledge of the target genes is still fragmentary. Nevertheless, Il-6 was found to be indirectly influenced by STAT3, which is the protein product of one of the target genes of miR-214-3p, while EEF1A1, a target of miR-30e-5p, directly regulates the transcription of the IFN-γ gene. Moreover, SQSTM1, DHX36 and PIK3R1 are involved in different cytokine signalling pathways. The next step of our study will be to analyse the pairs miRNA-target genes identified in the present in silico analysis.

## Conclusions

MSC and BL demonstrated slight differences in miRNA expression, regardless of SRLV infection. Our present findings lend support to our earlier hypothesis that the immune response in the udder is local and acts independently of the systemic immune response. We found that some miRNAs are not only involved in regulating goat immune gene expression in lentivirus infections, but also influence lactation processes, regardless of the health status of the host, and their expression does appear to be influenced by the metabolic burdens at the early lactation stage or at the lactation peak. Furthermore, some of the studied miRNAs may influence virus replication in the host cells by regulating the expression of their target genes. However, the identified target genes are capable of performing ambiguous roles, i.e. both protecting the host against virus infection and facilitating virus replication and host cell infection; furthermore, while some of them facilitate only one course of action, others facilitate both.

## Supplementary Information

Supplementary Figure S1.

Supplementary Figure S2.

Supplementary Figure S3.

Supplementary Figure S4.

Supplementary Figure S5.

Supplementary Table S1.

Supplementary Table S2.

Supplementary Table S3.

Supplementary Table S4.

Supplementary Table S5.

Supplementary Table S6.

Supplementary Table S7.

Supplementary Table S8.

Supplementary Table S9.

Supplementary Table S10.

Supplementary Table S11.

Supplementary Table S12.

Supplementary Table S13.

Supplementary Table S14.

Supplementary Table S15.

Supplementary Table S16.

Supplementary Table S17.

## Data Availability

All data generated or analysed during this study are included in this published article (Table S17).

## Abbreviations

APPs: Acute phase proteins
CAE: Caprine arthritis-encephalitis
CBX5: Chromobox protein homolog 5
CCNT2: Cyclin T2
CDC25A: M-phase inducer phosphatase 1
CDC25C: M-phase inducer phosphatase 3
CMV: Cytomegalovirus
CN1H1: Protein cornichon homolog 1
Cp: Ceruloplasmin
DDIT4: DNA damage-inducible transcript 4 protein
DENV: Dengue virus
DGAT2: Diacylglycerol O-acyltransferase 2
DHX36: ATP-dependent DNA/RNA helicase DHX36
EEF1A1: Elongation factor 1-alpha 1
FABP3: Transport of long-chain fatty acids
FASN: Fatty acid synthase
FIV: Feline immunodeficiency virus
GMEC: Goat mammary epithelial cells
H1HN1: Influenza A virus
HBV: Hepatitis B virus
HCV: Hepatitis C virus
HDGF: Hepatoma-derived growth factor
HHV-8: Human herpesvirus 8
HIV: Human immunodeficiency virus
HNRNPA1: Heterogeneous nuclear ribonucleoprotein A1
Hp: Haptoglobin
HPV16: Human papilloma virus 16
HSV-1: Herpes simplex virus 1
IFN-β: Interferon β
IGFR1: Insulin-like growth factor 1 receptor
IL-1α: Interleukin 1α
IL-1β: Interleukin 1β
Il-6: Interleukin 6
IL-8: Interleukin 8
INRA: Institut National de la Recherche Agronomique
LARP1: La-related protein 1
MAP3K5/ASK1: Mitogen-activated protein kinase 5 (apoptosis signal-regulating kinase 1)
MAPK: P38 mitogen-activated kinase
miRNA: MicroRNA
NCOA3: Nuclear receptor coactivator 3
NUCKS1: Nuclear ubiquitous casein and cyclin-dependent kinase substrate 1
PFI: Polish Fawn Improved
PIK3R1: Phosphatidylinositol 3-kinase regulatory subunit alpha
PKN2: Serine/threonine-protein kinase N2
PPARA: Peroxisome proliferator-activated receptor alpha
PTEN: Phosphatidylinositol 3,4,5-trisphosphate 3-phosphatase and dual-specificity protein phosphatase PTEN
PWI: Polish White Improved
RA: Rheumatoid arthritis
RAB7A: Ras-related protein Rab-7a
RANBP2: E3 SUMO-protein ligase RanBP2
ROCK2: Rho-associated protein kinase 2
RSV: Respiratory syncytial virus
SAA: Serum amyloid A
SARS-CoV: Severe acute respiratory syndrome-associated coronavirus
SCD5: Stearoyl-CoA desaturase 5
SIV: Simian immunodeficiency virus
SNAPIN: SNARE-associated protein Snapin
SPEN: Msx2-interacting protein
SQSTM1: Sequestosome 1
SRLV: Small ruminant lentivirus
SRLV-SN: SRLV-seronegative
SRLV-SP: SRLV-seropositive
STAT3: Signal transducer and activator of transcription 3
TNF-α: Tumour necrosis factor
UBN1: Ubinuclein-1
VACV: Vaccinia virus
VSV: Vesicular stomatitis virus
