# Supplementary material for: miRNA expression patterns in blood leukocytes and milk somatic cells of goats infected with small ruminant lentivirus (SRLV)
Source: Sci Rep. 2022 Aug 2;12:13239. doi: 10.1038/s41598-022-17276-y (PMC9344810; doi:10.1038/s41598-022-17276-y)
Supplement: Supplementary file 1 — Supplementary Figure S1. [file 41598_2022_17276_MOESM1_ESM.docx]

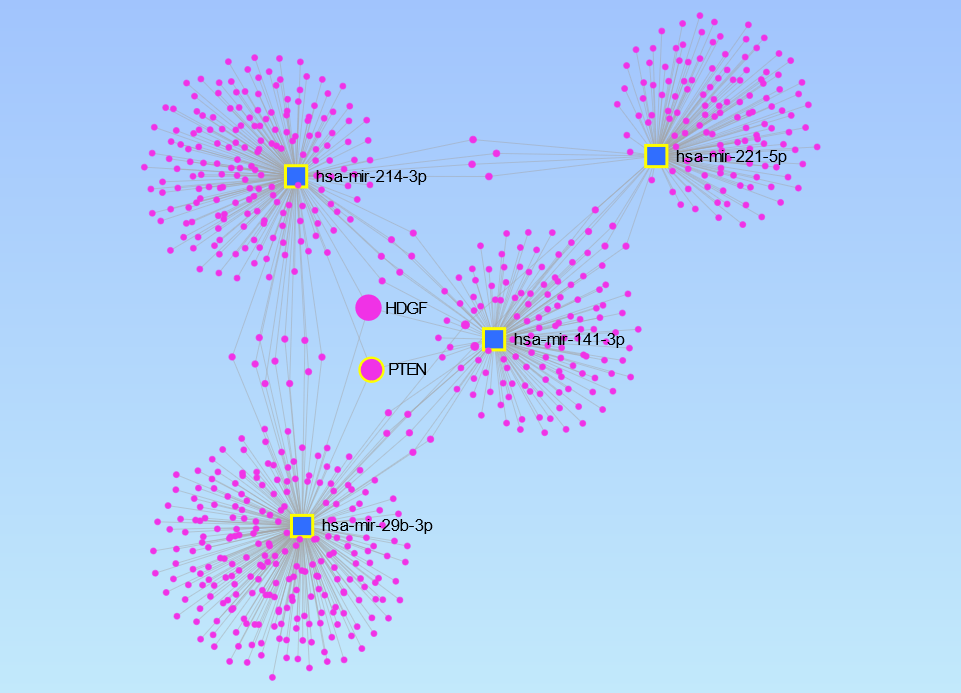


Figure S1. The relationship between four miRNAs: hsa-miR-29b-3p hsa-miR-214-3p, hsa-miR-221-5p, has-mir-141-3p, expressed in milk somatic cells, and their target genes identified using miRNet software
