# Supplementary material for: miRNA expression patterns in blood leukocytes and milk somatic cells of goats infected with small ruminant lentivirus (SRLV)
Source: Sci Rep. 2022 Aug 2;12:13239. doi: 10.1038/s41598-022-17276-y (PMC9344810; doi:10.1038/s41598-022-17276-y)
Supplement: Supplementary file 3 — Supplementary Figure S3. [file 41598_2022_17276_MOESM3_ESM.docx]

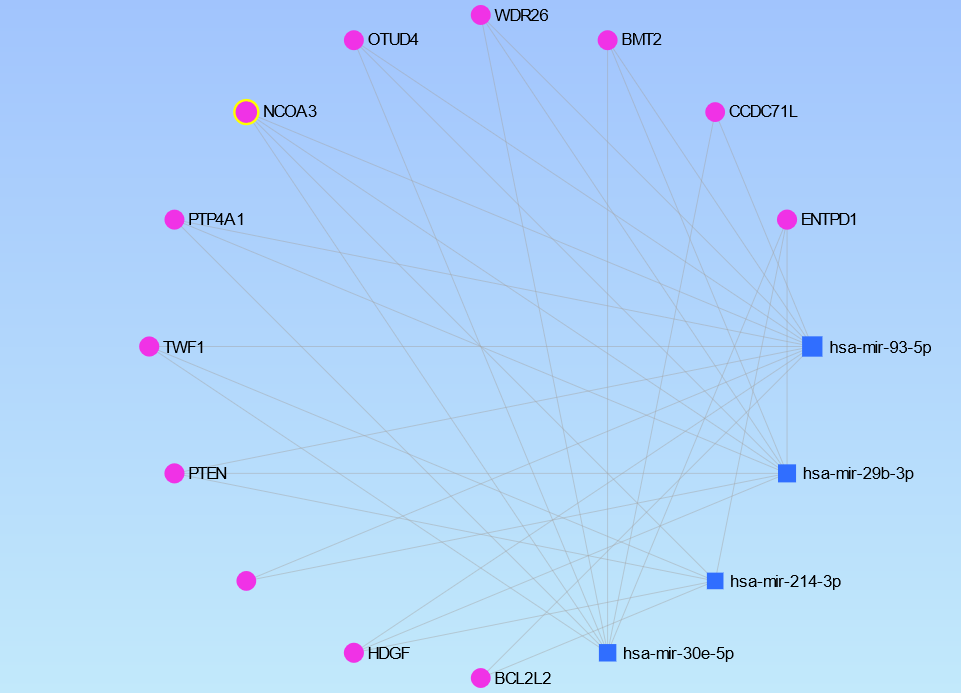


**Figure S3. The relationship between miR-29b-3p, miR-93-5p, and mir-214-3p, mir-30e-5p, expressed in blood leukocytes, and their target genes, common at least for three miRNAs, identified using miRNet software**
