# Supplementary material for: miRNA expression patterns in blood leukocytes and milk somatic cells of goats infected with small ruminant lentivirus (SRLV)
Source: Sci Rep. 2022 Aug 2;12:13239. doi: 10.1038/s41598-022-17276-y (PMC9344810; doi:10.1038/s41598-022-17276-y)
Supplement: Supplementary file 5 — Supplementary Figure S5. [file 41598_2022_17276_MOESM5_ESM.docx]

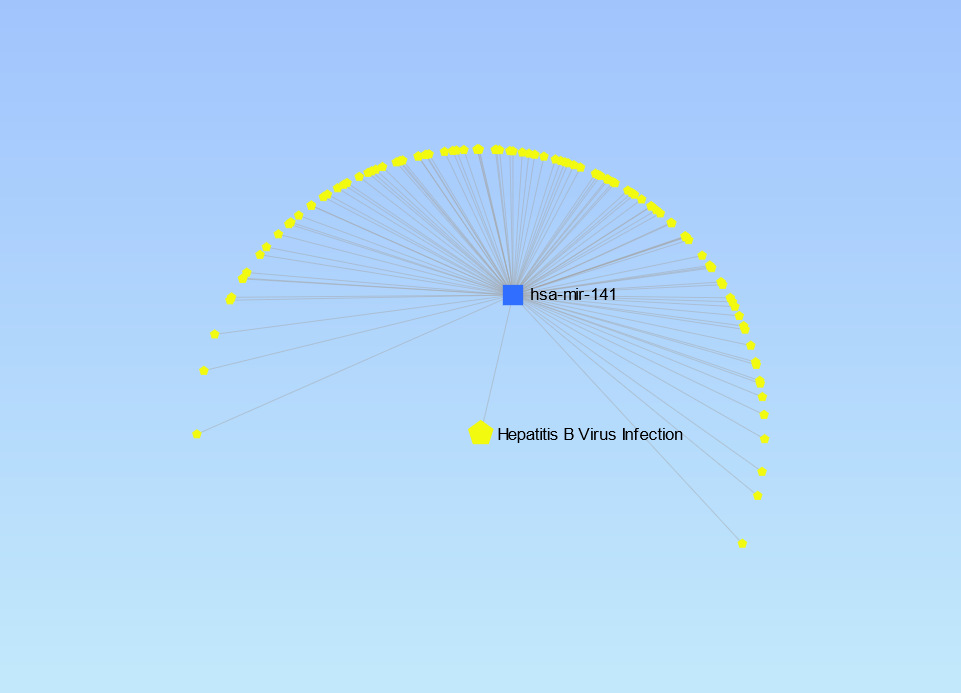


**Figure S5. The relationship between has-mir-141-3p, expressed in milk somatic cells and diseases emphasising viral infection, identified using miRNet software**
