# Supplementary material for: miRNA expression patterns in blood leukocytes and milk somatic cells of goats infected with small ruminant lentivirus (SRLV)
Source: Sci Rep. 2022 Aug 2;12:13239. doi: 10.1038/s41598-022-17276-y (PMC9344810; doi:10.1038/s41598-022-17276-y)
Supplement: Supplementary file 6 — Supplementary Table S1. [file 41598_2022_17276_MOESM6_ESM.docx]

Table S1. The average somatic cell count (SCC) with standard deviation (SD) in milk regarding time of sampling.

| Day of sampling after kidding | Average | SD |
| --- | --- | --- |
| 1 – just after kidding | 313,000 | 256,000 |
| 30 – early lactation | 369,000 | 241,000 |
| 60 – peak of lactation | 433,000 | 310,000 |
| 140 – mid-lactation | 546,000 | 383,000 |
| 200 – late lactation | 620,000 | 606,000 |
