# Supplementary material for: miRNA expression patterns in blood leukocytes and milk somatic cells of goats infected with small ruminant lentivirus (SRLV)
Source: Sci Rep. 2022 Aug 2;12:13239. doi: 10.1038/s41598-022-17276-y (PMC9344810; doi:10.1038/s41598-022-17276-y)
Supplement: Supplementary file 7 — Supplementary Table S2. [file 41598_2022_17276_MOESM7_ESM.docx]

**Table S2.** **Functional analysis of miRNAs using human genome target gene databases limited to immunity-related, antiviral response or viral processes genes.**

| **miRNA** | **Target gene – recommended name (GeneCards)** | **Target gene symbol** | **Biological function of the protein encoded by the target gene*** (abbreviations defined under the table) |
| --- | --- | --- | --- |
| **miR-214-3p** | Signal transducer and activator of transcription 3 | *STAT3* | - Is involved in biological processes such as viral processes (GO:0016032), keyword: host-virus interaction (KW-0945) - Is activated through phosphorylation **in response to various cytokines and growth factors** including IFNs, EGF, IL5, IL6, HGF, LIF and BMP2 - Is involved in **IL6 and related cytokines** induced signal transduction by JAK kinases - **Decreases** JFH-1 genotype 2a HCV infection after direct virus infection - Interacts with Latent nuclear antigen, a protein of HHV-8 |
|  | La-related protein 1 | *LARP1* | - **Positively regulates** the replication of DENV (Biologiocal process: positive regulation of viral genome replication (GO:0045070) - **Increased** VACV infection - **Decreased** influenza A H1N1 (A/Hamburg/04/2009) virus numbers - **Decreased** influenza A H1N1 (A/WSN/33) virus numbers - **Decreased** influenza A/WSN/33 replication |
|  | Protein cornichon homolog 1 | *CNIH1* | - Diseases associated with CNIH1 include Bacterial Conjunctivitis and Zika Fever. - **Increased** VACV infection |
|  | GTPase NRas | *NRAS* | - **Increased** VACV infection |
| **miR-221-5p** | Heterogeneous nuclear ribonucleoprotein A1 | *HNRNPA1* | - May play a role in HCV RNA replication; interacts with HCV NS5B and with the 5'-UTR and 3'-UTR of HCV RNA - **Increased** VACV infection - Colocalizes with HCV NS5B at speckles in the cytoplasm in a HCV-replication dependent manner. - Cleavage by Enterovirus 71 protease 3C results in increased translation of apoptosis protease activating factor APAF1, leading to apoptosis - Involved in biological processes such as viral processes (GO:0016032), keyword: host-virus interaction (KW-0945) |
| **miR-93-5p** | Mitogen-activated protein kinase kinase kinase 5 | *MAP3K5* | - MAP3K5/ASK1 complex is required for the innate immune response, which is essential for host defense against a wide range of pathogens - **Increased** VACV infection - Is involved in biological processes such as viral processes (GO:0016032), keyword: host-virus interaction (KW-0945) |
|  | Signal transducer and activator of transcription 3 | *STAT3* | - As above |
|  | Sequestosome 1 | *SQSTM1* | - May be involved in cell differentiation, apoptosis, immune response and regulation of K(+) channels - Is involved in **Il-1 signaling pathway** and **Cytokine Signaling** in Immune system - Interacts binarly with vif [P12504], a protein from Human immunodeficiency virus type 1 (HIV-1) group M subtype B (isolate NY5) |
|  | Serine/threonine-protein kinase N2 | *PKN2* | - Phosphorylates HCV NS5B leading to **stimulation of HCV RNA replication** - Colocalizes with HCV NS5B at perinuclear region in the cytoplasm. - Involved in biological process – a viral RNA genome replication (GO:0039694) |
|  | Rho-associated protein kinase 2 | *ROCK2* | - **Decreased** JFH-1 genotype 2a HCV infection, both after direct virus infection and viral supernatant infection. - **Increases** VACV infection - Is involved **in chemokine signaling pathway** |
|  | Chromobox protein homolog 5 | *CBX5* | - Interacts with John Cunningham virus (Human polyomavirus 2) agnoprotein; this interaction induces the dissociation of CBX5 from LBR (lamin-B receptor), resulting in destabilization of the host cell’s nuclear envelope. - Is involved in biological processes such as viral processes (GO:0016032), keyword: host-virus interaction (KW-0945) |
| **miR-30e-5p** | BCL2/Adenovirus E1B 19 KDa Protein-Interacting Protein 3-Like | *BNIP3L* | - Interacts with human adenovirus-2 E1B 19 kDa protein - Interacts with viral and cellular anti-apoptosis proteins. - Is involved in biological processes such as defense response to virus (GO:0051607), and viral processes (GO:0016032), keyword: host-virus interaction (KW-0945) |
|  | Beclin-1 | *BECN1* | - **Protects against infection** by a neurovirulent strain of Sindbis virus - May play a role in **antiviral host defense** - Interacts with HCMV/HHV-5 protein TRS1 - Interacts with murine gammaherpesvirus 68 M11 - Interacts with HHV-1 protein ICP34.5 - The cleavage is proposed to be an determinant to switch from autophagy to apoptosis pathways affecting cellular homeostasis including viral infections and survival of tumor cells |
|  | ATP-dependent DNA/RNA helicase DHX36 | *DHX36* | - Plays role as a sensor to initiate **antiviral responses** - As a component of a multi-helicase-TICAM1 complex acts as a cytoplasmic sensor of viral double-stranded RNA (dsRNA) - Plays a role in the activation of a cascade of antiviral responses including the **induction of pro-inflammatory cytokines** via the adapter molecule TICAM1 - Is involved in biological process – defense response to virus (GO:0051607), keyword antiviral defence (KW-0051) |
|  | DNA damage-inducible transcript 4 protein | *DDIT4* | - Is required for mTORC1-mediated **defense against viral protein synthesis** and virus replication (By similarity) - Is involved in biological processes such as defense response to virus (GO:0051607) |
|  | Homeodomain-interacting protein kinase 2 | *HIPK2* | - **Increased** HCMV strain AD169 replication - **Increased** VACV infection |
|  | Ras-related protein Rab-7a | *RAB7A* | - Plays important roles in participating in the life cycle of viruses - **Decreases** HCV replication - **Decreases** HPV 16 (HPV16) pseudovirus infection - **Increased** VACV infection - **Positively regulates viral process** (GO:0048524), involved in processes such as viral releasing from host cell (GO:0019076), keyword: host-virus interaction (KW-0945) |
|  | E3 SUMO-protein ligase RanBP2 | *RANBP2* | - Is implicated in the nuclear delivery and integration of certain human viruses, including Herpes simplex and HIV-1 - **Increased** VACV infection - Is involved in biological processes such as intracellular transport of virus (GO:0075733), viral process (GO:0016032), and viral transcription (GO:0019083) - Is involved in **ISG15 antiviral mechanism** (Enzyme and pathway databases Reactome R-HSA-1169408) |
|  | SNARE-associated protein Snapin | *SNAPIN* | - Interacts with human cytomegalovirus/HHV-5 protein UL70. - Affects viral DNA replication (decreasing the level of Snapin via specific small interfering RNAs **decreased the number of viral DNA copies** and titer in HCMV-infected U373-S cells) - **Increases** HPV18 LCR reporter activity - Interacts with HCMV pUL130. - HIV‐1 preferentially localizes with Snapin following entry to Dendritic Cells as a mechanism to evade immune detection - Is involved in biological processes such as viral processes ( GO:0016032) keyword: host-virus interaction (KW-0945) |
|  | TATA box-binding protein-like protein 1 | *TBPL1* | - Among its related pathways are Human T-cell leukemia virus 1 infection - **Increased** VACV infection |
|  | Cullin 2 | *CUL2* | - Interacts with HRSV protein NS1 - Involved in biological processes such as viral processes (GO:0016032), keyword: host-virus interaction (KW-0945) - Interacts binarly with vif [P12504], a protein from HIV-1 1 group M subtype B (isolate NY5). |
|  | G1/S-specific cyclin-E2 | *CCNE2* | - Activated by papilloma viral oncoproteins E6 and E7 which bind to and inactivate p53 and Rb - **Increased** VACV infection |
|  | Cyclin T2 | *CCNT2* | - the p-TEFb complex containing this cyclin interacts with, and act as a negative regulator of HIV-1 Tat protein - Promotes transcriptional activation of early and late herpes simplex virus 1/HHV-1 promoters - Downregulates CCNT2-mediated activation of viral promoters during herpes simplex virus 1/HHV-1 infection (PubMed:21509660). - Interacts with HIV-2 and SIV Tat. - Does not bind efficiently to the transactivation domain of the HIV-1 Tat - Involved in biological processes such as early viral transcription (GO:0019085), late viral transcription (GO:0019086), keyword: host-virus interaction (KW-0945) |
|  | Elongation factor 1-alpha 1 | *EEF1A1* | - **Decreases** influenza A H1N1 (A/Hamburg/04/2009) virus numbers - **Decreases** influenza A/WSN/33 replication - With PARP1 and TXK, forms a complex that acts as a T helper 1 (Th1) cell-specific transcription factor and **binds the promoter of** **IFN-gamma** to directly regulate its transcription, and is thus involved importantly in Th1 cytokine production |
|  | Nuclear ubiquitous casein and cyclin-dependent kinase substrate 1 | *NUCKS1* | - **Increased** VACV infection - Enhances HIV-1 Tat-mediated transcriptional activation on the HIV-1 LTR (knockdown of NUCKS1 by siRNA significantly reduces Tat-mediated transcriptional activation) - NUCKS1, both ectopically expressed and endogenous in HEK293 cells, interacts directly with HIV-1 Tat - Its overexpression moderately **induces HIV-1 production** with increased release of p24 in latently infected cells - Is involved in biological processes such as - modulation by host of viral RNA-binding transcription factor activity (GO:1990969), positive regulation by host of viral genome replication (GO:0044829), positive regulation by host of viral transcription (GO:0043923), positive regulation by host of viral genome replication (GO:0044829), and release from viral latency (GO:0019046) |
|  | Msx2-interacting protein | *SPEN* | - Interacts with EBV BSFL2/BMLF1 - Increased VACV infection - **Decreases** HIV-1 infection - Involved in biological processes such as viral processes (GO:0016032), keyword: host-virus interaction (KW-0945) |
|  | Ubinuclein-1 | *UBN1* | - Interacts with EBV BZLF1; Its overexpression represses the EBV productive cycle whereas its downregulation by short hairpin RNA (shRNA) increases virus production. Ubn-1 blocks EB1-DNA interaction - **Increased** VACV infection - Is involved in biological processes such as viral processes (GO:0016032), keyword: host-virus interaction (KW-0945) |
|  | Vimentin | *VIM* | - Bacterial and viral pathogens have been shown to attach to this protein on the host cell surface. - **Decreased** HCV replication - **Increased** VACV infection - One of the KEGG pathways for VIM Gene is “Epstein-Barr virus infection” - Is involved in biological processes such as viral processes (GO:0016032), keyword: host-virus interaction (KW-0945) - Interacts with: - non-structural protein 4A (PRO_0000037966) from DENV type 2 (strain Thailand/NGS-C/1944). - Core protein p21 (PRO_0000037566) from HCV genotype 1a (isolate H) |
| **miR-29b-3p** | Complement component C1q receptor | CD93 | - Interacts with HCV/HCV core protein - **Increased** VACV infection - **Decreased** viability after SINV dsTE12Q infection - **Decreased** SINV capsid and autophagosome LC3 protein colocalization - Is involved in biological processes such as viral processes (GO:0016032), keyword: host-virus interaction (KW-0945) |
|  | ATP-dependent RNA helicase DDX3X | DDX3X | - Is involved in innate immunity, acting as a viral RNA sensor. Binds viral RNAs and promotes the production of type I interferon (**IFN-alpha and IFN-beta**). Potentiate MAVS/DDX58-mediated induction of **IFNB** in early stages of infection - Is thought to be involved in viral replication in the cytoplasm - Specifically promotes translation of a subset of viral and cellular mRNAs carrying a 5'proximal stem-loop structure in their 5'UTRs - Can bind to viral RNAs and via association with MAVS/IPS1 and DDX58/RIG-I is thought to induce signaling in early stages of infection. - Is involved in regulation of apoptosis - Appears to be a prime target for viral manipulations - Interacts with HBV polymerase in the cytoplasm; this interaction may inhibit DDX3X interaction with the IKBKE/TBK1 complex, and hence impair IKBKE/TBK1-mediated increase in IFNB production (HBV polymerase and possibly VACV protein K7 inhibit IFNB induction probably by dissociating DDX3X from TBK1 or IKBKE) - **Facilitates** **HCV replication**. During infection, HCV core protein inhibits the interaction between MAVS and DDX3X and therefore **impairs MAVS-dependent INFB induction** and might recruit DDX3X to HCV replication complex - Directly interacts with HCV core protein in the cytoplasm. HCV core protein inhibits the IPS1-dependent function in viral RNA sensing and may switch the function from a **INFB inducing** to a HCV replication mode - Interacts (via C-terminus) with MAVS/IPS-1; this interaction occurs rapidly, but transiently after **Sendai virus** infection. The interaction potentiates MAVS-mediated **IFNB** induction - Is involved in HIV-1 replication. Acts as a cofactor for XPO1-mediated nuclear export of incompletely spliced HIV-1 Rev RNAs - It is found to be induced upon virus infection - Interacts with VACV protein K7 - **Facilitates** ZIKV replication. - **Facilitates** DENV replication - **Facilitates** VEEV replication. Interacts with VEEV non-structural protein 3 - Is involved in biological processes such as viral processes (GO:0016032), keyword: host-virus interaction (KW-0945); positive regulation of viral genome replication (GO:0045070), and response to virus (GO:0009615) - Is involved in post-transcriptional modification: is phosphorylated by IKBKE at Ser-102 after ssRNA viral infection - Interacts binarly with: - K7R [P68467] from VACV (strain Copenhagen) - tat [P04608] from HIV-1 group M subtype B (isolate HXB2) - Trpm7 [Q923J1] from Mus musculus - VACWR039 [P68466] from VACV (strain Western Reserve) - Core protein p21 (PRO_0000037517) from HCV genotype 1a (isolate 1) - Core protein p21 (PRO_0000037566) from HCV genotype 1a (isolate H) - Core protein p21 (PRO_0000037559) from HCV (isolate Glasgow) - Q99IB8 from HCV genotype 2a (isolate JFH-1) - Core protein p21 (PRO_0000037541) from HCV genotype 1b (isolate Con1). |
|  | F-box/WD repeat-containing protein 7 | FBXW7 | - Increased VACV infection - Its ectopic expression could strongly down-regulate NS5B level during HCV replication and consequently it inhibited the virus replication - Is involved in biological processes such as viral processes (GO:0016032), keyword: host-virus interaction (KW-0945) - Interacts binarly with Q91LX9, a protein from HHV 8. |
|  | Transcription initiation factor TFIID subunit 5 | TAF5 | - Is involved in VACV infection - **Increased** SINV infection - Is involved in biological processes such as viral processes (GO:0016032), keyword: host-virus interaction (KW-0945) |
|  | TATA-Box Binding Protein Associated Factor 11 | TAF11 | - **Decreased** HPV16 pseudovirus infection - **Increased** VACV infection - **Increased** replication of VACV - Is involved in biological process: positive regulation by host of viral transcription (GO:0043923) |
|  | Vacuolar Protein Sorting-Associated Protein 37C | VPS37C | - **Increased** VACV infection - Among its related pathways are Budding and maturation of HIV virion and HIV Life Cycle (biological processes: viral budding via host ESCRT complex (GO:0039702), and viral life cycle (GO:0019058)) (facilitate virion budding from the host) - Creates cytoplasm filled vesicular bodies and therefore is involved in: Budding and maturation of HIV virion (With the virus components precariously assembled on the inner leaflet of the plasma membrane, the host cell machinery is required for viral budding. The virus takes advantage of the host ESCRT pathway to terminate Gag polymerization and catalyze release (Reactome R-HSA-162588)) |
|  | AP-1 complex subunit gamma-1 | AP1G1 | - Is involved in VACV infection - **Decreased** viability after Maraba virus infection - Involved in HCMV infection and HIV-1 infection pathways - Is involved in biological process: regulation of defense response to virus by virus GO:0050690) |
|  | Caveolin 2 | CAV2 | - **Increased** VACV infection - Is involved in biological processes such as positive regulation by host of viral process (GO:0044794), positive regulation by host of viral release from host cell (GO:0044791), and receptor-mediated endocytosis of virus by host cell (GO:0019065) |
|  | Cyclin-T2 | CCNT2 | - The p-TEFb complex containing this cyclin was reported to interact with, and act as a **negative regulator** of HIV-1 Tat protein - Among its related pathways are Formation of HIV-1 elongation complex. - Promotes transcriptional activation of early and late herpes simplex virus 1/HHV-1 promoter containing HIV-1 Tat and HIV Life Cycle. - Interacts with HIV-2 and SIV Tat. Does not bind efficiently to the transactivation domain of the HIV-1 Tat - Is involved in biological processes such as early viral transcription (GO:0019085), late viral transcription (GO:0019086), keyword: host-virus interaction (KW-0945) - Interacts with MON1B; downregulates CCNT2-mediated activation of viral promoters during herpes simplex virus 1/HHV-1 infection |
|  | Dynein light chain Tctex-type 1 | DYNLT1 | - Interacts with viral proteins, like the minor capsid protein L2 of HPV; this interaction is essential for virus intracellular transport during entry - Is required for dynein-mediated delivery of the viral nucleic acid to the host nucleus - Is involved in intracellular targeting of D-type retrovirus gag polyproteins to the cytoplasmic assembly site - Is involved in biological processes such as intracellular transport of viral protein in host cell (GO:0019060), viral entry into host cell (GO:0046718), transport of viral material towards nucleus (GO:0075606), keyword: Cytoplasmic inwards viral transport (KW-1176), viral process (GO:0016032), microtubule-dependent intracellular transport of viral material towards nucleus (GO:0075521), and viral entry into host cell (GO:0046718) - Interacts with Mason-Pfizer monkey virus protein Gag |
|  | Phosphatidylinositol 3-kinase regulatory subunit alpha | PIK3R1 | - Interacts with HIV-1 Nef to activate the Nef associated p21-activated kinase (PAK). This interaction depends on the C-terminus of both proteins and leads to increased production of HIV - Interacts with HCV NS5A - Increased VACV infection - Interacts with herpes simplex virus 1 UL46 and varicella virus ORF12; this interaction activates the PI3K/AKT pathway - Is involved in biological processes such as viral processes (GO:0016032), keyword: host-virus interaction (KW-0945) - Interacts binarly with: - NS [P03496], a protein from Influenza A virus (strain A/Puerto Rico/8/1934 H1N1). - VP3 [Q99152], a protein from Chicken anemia virus (isolate Germany Cuxhaven-1). - P0DOJ9 a protein from Mus musculus polyomavirus 1 - Is involved in RET signalling pathway (Interleukin receptor SNC signalling; Interleukin-3, 5 and GM-CSF signaling; Interleukin-2 signaling, IGF1R signaling cascade) - Is involved in Il-2 pathway - Is involved in Common Cytokine Receptor Gamma-Chain Family Signaling Pathways (Il-21 signaling pathway; Il-7 Signalig patjway; - Il-9 signaling pathway (Common Cytokine Receptor Gamma-Chain Family Signaling Pathways; IL-15 Signaling Pathways; IL-2 Signaling Pathways) |
|  | BTB/POZ domain-containing protein KCTD5 | KCTD5 | - Interacts with AAV-2 REP proteins - Increased VACV infection - Is involved in biological processes such as viral processes (GO:0016032), keyword: host-virus interaction (KW-0945) - Interacts binarly with: - with AAV-2 REP proteins - Rep68 [P03132] from AAV-2 (isolate Srivastava/1982) |
|  | Cytochrome C | CYCS | - **Decreased** HIV-LTR-beta-galactosidase protein expression - Resistant to VACV-A4L infection - Involved in “HIV-1 Nef- Negative effector of Fas and TNF-alpha0.46” and “Apoptotic Pathways Triggered By HIV1” pathways |
|  | N-myc proto-oncogene protein | MYCN | - **Increased** VACV infection |
|  | Nuclear factor 1 A-type NFIA Antisense RNA 1 | NFIA | - Recognizes and binds the palindromic sequence 5'-TTGGCNNNNNGCCAA-3' present in viral and cellular promoters and in the origin of replication of adenovirus type 2; These proteins are individually capable of activating transcription and replication - Is involved in biological process: viral genome replication (GO:0019079) |

Databases used: DIANA-tools – TarBase v8.0 (<http://diana.imis.athena-innovation.gr/DianaTools/index.php?r=tarbase/index>**;** NCBI (<https://www.ncbi.nlm.nih.gov/pmc/articles/PMC5753203/>); The Database for Annotation, Visualization and Integrated Discovery (DAVID) v6.8 (https://david.ncifcrf.gov/tools.jsp ). UniProtKB/Swiss-Prot database ([https://www.uniprot.org/statistics/Swiss-Prot - UniProtKB/Swiss-Prot UniProt release 2020_03](https://www.uniprot.org/statistics/Swiss-Prot%20-%20UniProtKB/Swiss-Prot%20UniProt%20release%202020_03), GeneCards (https://www.genecards.org/); Reactome database (<https://reactome.org/>), access Apr-22, 2020;

AAV-2 – adeno-associated virus 2, AKT gene encodes RAC-alpha serine/threonine-protein kinase, APAF1 – apoptosis protease activating factor, ASK1 – Apoptosis signal-regulating kinase 1 also known as mitogen-activated protein kinase 5, BMP2 – Bone Morphogenetic Protein 2, CBX5 – Chromobox 5, DENV – dengue virus, dsRNA – double-stranded RNA, EBV – Epstein-Barr virus, ESCRT-III – endosomal sorting required for transport complex III, EGF – epidermal growth factor HBV – Hepatitis B Virus, HCV – Hepatitis C Virus, HCMV – human cytomegalovirus, HGF – Hepatocyte growth factor, HHV – Human herpesvirus, HIV-1 – Human Immunodeficiency Virus Type 1, HPV – Human Papilloma Virus, HRSV – human respiratory syncytial virus, IFN – interferon, IKBKE – Inhibitor Of Nuclear Factor Kappa B Kinase Subunit Epsilon, Il – interleukin, LBR – lamin-B receptor, MAVS – Mitochondrial Antiviral Signaling Protein, mTORC1 – mammalian target of rapamycin complex 1, PARP1 – Poly(ADP-Ribose) Polymerase 1, PI3Ks – Phosphoinositide 3-kinases also called phosphatidylinositol 3-kinases, P-TEFb – positive transcription elongation factor, TICAM1 – Toll Like Receptor Adaptor Molecule 1, TXK – Tyrosine Kinase; VACV – vaccinia virus, VEEV – Venezuelan equine encephalitis virus, ZIKV – Zika virus
