# Supplementary material for: miRNA expression patterns in blood leukocytes and milk somatic cells of goats infected with small ruminant lentivirus (SRLV)
Source: Sci Rep. 2022 Aug 2;12:13239. doi: 10.1038/s41598-022-17276-y (PMC9344810; doi:10.1038/s41598-022-17276-y)
Supplement: Supplementary file 8 — Supplementary Table S3. [file 41598_2022_17276_MOESM8_ESM.docx]

Table S3. The Biological Processes according to the Gene Ontology (GO) and GO annotation (<https://www.ebi.ac.uk/QuickGO/term/>) and the UniProt base: Category - Biological process (KW-9999) (https://www.uniprot.org/keywords/)

|  | Access No. | Biological process | Definition/key words –definition/exact synonyms/comments |
| --- | --- | --- | --- |
| 1 | GO:  0009615 | response to virus | Any process that results in a change in state or activity of a cell or an organism (in terms of movement, secretion, enzyme production, gene expression, etc.) as a result of a stimulus from a virus. |
| 2 | GO:  0016032 | viral process | A multi-organism process in which a virus is a participant. The other participant is the host. Includes infection of a host cell, replication of the viral genome, and assembly of progeny virus particles. In some cases the viral genetic material may integrate into the host genome and only subsequently, under particular circumstances, 'complete' its life cycle.  **Exact synonyms:** virus process; viral infection  **Comments:** See also the biological process terms 'viral infectious cycle; GO:0019058' and 'lysogeny; GO:0030069'.  **Key-word*:** Host-virus interaction (KW-0945):  Viral or cellular protein involved in a host-virus interaction. Viruses interact with many cellular pathways to achieve their replication cycle. Entry into the host cell, transport to the viral replication sites or viral exit from the host cell are all steps that require specific interactions between the virus and its host. Additionally, the evasion from the host immune response requires a lot of viral proteins to associate with and inhibit cellular proteins with antiviral functions. |
| 3 | GO:  0019046 | release from viral latency | The process by which a virus begins to replicate following a latency replication decision (switch) |
| 4 | GO:0019048 | modulation by virus of host process | The process in which a virus effects a change in the structure or processes of its host organism  **Exact synonyms:** viral interaction with host, virus-host process, modulation by virus of host anatomy or process, host-virus interaction |
| 5 | GO:  0019058 | viral life cycle | A set of processes which all viruses follow to ensure survival; includes attachment and entry of the virus particle, decoding of genome information, translation of viral mRNA by host ribosomes, genome replication, and assembly and release of viral particles containing the genome |
| 6 | GO:  0019060 | intracellular transport of viral protein in host cell | The directed movement of a viral protein within the host cell  **Exact synonyms:** intracellular transport of viral proteins in host cell; intracellular viral protein transport  **Comments:** This term is for annotation of proteins responsible for the movement of individual viral proteins, rather than the whole viral particle. |
| 7 | GO:  0019065 | receptor-mediated endocytosis of virus by host cell | Any receptor-mediated endocytosis that is involved in the uptake of a virus into a host cell; successive instances of virus endocytosis result in the accumulation of virus particles within the cell.  **Exact synonyms:** receptor-mediated endocytosis of virus by host; virus receptor-mediated endocytosis by host; viral entry into host cell via receptor-mediated endocytosis; receptor mediated endocytosis of virus by host; receptor mediated endocytosis of virus particle by host; receptor mediated endocytosis by host of virus particle; viral receptor mediated endocytosis |
| 8 | GO:  0019076 | viral release from host cell | The dissemination of mature viral particles from the host cell, e.g. by cell lysis or the budding of virus particles from the cell membrane  **Exact synonyms**: release of virus from host; virus exit from host cell; viral release; viral exit; viral shedding |
| 9 | GO:  0019079 | viral genome replication | Any process involved directly in viral genome replication, including viral nucleotide metabolism |
| 10 | GO:  0019083 | viral transcription | The process by which a viral genome, or part of a viral genome, is transcribed within the host cell.  **Exact synonym:** viral genome expression |
| 11 | GO:  0019085 | early viral transcription | The first phase of viral transcription that occurs after entry of the virus into the host cell, but prior to viral genome replication. It involves the transcription of genes for non-structural proteins, and for lytic viruses, the early gene products are involved in establishing control over the host cell. |
| 12 | GO:  0019086 | late viral transcription | The transcription of the final group of viral genes of the viral life cycle, following middle transcription, or where middle transcription doesn't occur, following early transcription. Involves the transcription of genes encoding structural proteins |
| 13 | GO:  0030683 | mitigation of host immune response by virus | A process by which a virus avoids the effects of the host organism's immune response. The host is defined as the larger of the organisms involved in a symbiotic interaction.  **Exact synonyms**: negative regulation by virus of intracellular antiviral response; viral inhibition of intracellular antiviral response; suppression of host intracellular antiviral response by virus; suppression by virus of host extracellular antiviral response; suppression of host extracellular antiviral response by virus; suppression by virus of host intracellular antiviral response; negative regulation by virus of extracellular antiviral response; negative regulation of host extracellular antiviral response by virus; inhibition of extracellular antiviral response; negative regulation of host intracellular antiviral response by virus; mitigation by virus of host immune response |
| 14 | GO:  0039694 | viral RNA genome replication | The replication of a viral RNA genome |
| 15 | GO:  0039702 | viral budding via host ESCRT complex | Viral budding which uses a host ESCRT protein complex, or complexes, to mediate the budding process |
| 16 | GO:0  043923 | positive regulation by host of viral transcription | Any process in which a host organism activates or increases the frequency, rate or extent of viral transcription, the synthesis of either RNA on a template of DNA or DNA on a template of RNA.  **Exact synonym:** positive regulation of viral transcription by host |
| 17 | GO:  0044791 | positive regulation by host of viral release from host cell | A process in which a host organism activates or increases the frequency, rate or extent of the release of a virus with which it is infected, from its cells |
| 18 | GO:  0044829 | positive regulation by host of viral genome replication | A process in which a host organism activates or increases the frequency, rate or extent of viral genome replication |
| 19 | GO:  0045070 | positive regulation of viral genome replication | Any process that activates or increases the frequency, rate or extent of viral genome replication  **Exact synonym**: up regulation of viral genome replication |
| 20 | GO:  0046718 | viral entry into host cell | The process that occurs after viral attachment by which a virus, or viral nucleic acid, breaches the plasma membrane or cell envelope and enters the host cell. The process ends when the viral nucleic acid is released into the host cell cytoplasm.  **Keyword - Virus entry into host cell (KW-1160)**  Viral protein involved in the virion entry into a host cell. Entry is a multistep process that mostly requires binding to the target cell, penetration into the host cell cytoplasm, intracellular transport of viral components and genome release to the replication site of the virus  **Exact synonyms:** phage translocation; virus entry into host cell; virion penetration into host cell; virion penetration entry of virus into host cell; viral penetration  **Comments:** Viral attachment to the host cell is not part of viral entry in GO because virus attachment does not always lead to viral entry: attachment can also result in the virion being carried by the host cell to another location. |
| 21 | GO:  0048524 | positive regulation of viral process | Any process that activates or increases the frequency, rate or extent of a multi-organism process in which a virus is a participant |
| 22 | GO:  0050690 | regulation of defense response to virus by virus | Any viral process that modulates the frequency, rate, or extent of the antiviral response of the host cell or organism  **Exact synonym**: regulation of antiviral response |
| 23 | GO:  0051607 | defense response to virus | Reactions triggered in response to the presence of a virus that act to protect the cell or organism  **Keyword - Antiviral defense (KW-0051):**  Protein synthesized or activated in the cell in response to viral infection, or protein with specific antiviral activity within the cell. Eukaryotic cells have an innate immune mechanism to fight viral infection, which is activated through the interferon signaling pathway or through dsRNA detection in the cytoplasm. It leads to the establishment of an antiviral cell state, which prevents virus replication or induces apoptosis. Most viruses have developed specific proteins to prevent the establishment of an antiviral state. About half of all bacteria and most archaea have a CRISPR (clustered regularly interspersed short plaindromic repeats) system of adaptive immunity to exogenous DNA. CRISPRs clusters are tandem arrays of alternating repeats and spacers, where the spacers in some cases are homologous to sequences from virus and plasmid genomes. The CRISPR arrays are transcribed, processed and in some way aid in detection and resistance to foreign DNA. In at least a few bacteria (*Escherichia coli*, *Staphylococcus epidermidis*) it seems DNA is the target, whereas in *Pyrococcus furiosis* it seems the CRISPR system targets RNA |
| 24 | GO:  0075521 | microtubule-dependent intracellular transport of viral material towards nucleus | The directed movement of a virus, or part of a virus, towards the host cell nucleus using host microtubules.  **Keyword - Microtubular inwards viral transport (KW-1177):**  Viral protein that allows the active transport of complete particles and viral components along microtubules toward the intracellular replication sites during virus entry. This transport, which usually involves motor proteins like dynein or polymerization/depolymerization reactions as a driving force, is mostly used by viruses that replicate their genome near or in the nucleus. Neurotropic viruses for example, often enter neurons at the terminal axon and their viral genome must be moved retrogradely to cell bodies. Viruses such as adenovirus, Adeno-associated virus, rabies virus, canine parvovirus, vaccinia, foamy virus, human papillomavirus 16 and herpes virus utilize this type of intracellular transport  **Exact synonyms:** microtubule-dependent intracellular transport of viral material to nucleus |
| 25 | GO:  0075606 | transport of viral material towards nucleus | The directed movement of a virus, or part of a virus, towards the host cell nucleus. The process begins after viral entry, and ends when the viral material is at the nuclear membrane.  **Keyword - Cytoplasmic inwards viral transport (KW-1176):**  Viral protein that allows the active transport of viral components along cytoskeletal filaments toward the intracellular replication sites during virus entry. Viruses such as adenoviruses, adeno-associated virus, vaccinia virus, poliovirus, canine parvovirus, African swine fever virus, rabies virus, human herpes virus 1, foamy virus are thought to use active intracellular transport of viral components  **Exact synonyms:** cytoplasmic inwards viral transport; viral genome transport to host cell nucleus; transport of viral material to nucleus  **Comments:** This process does not include the viral material crossing the nuclear membrane. For transport of viral material into the nucleus, consider instead: 'viral penetration into host nucleus ; GO:0075732'. |
| 26 | GO:  0075733 | intracellular transport of virus | The directed movement of a virus, or part of a virus, within the host cell  **Exact synonyms:** movement of virus within host cell; egress of virus within host cell; intracellular transport of viral material; viral genome transport in host cell; viral egress |
| 27 | GO:  1990969 | modulation by host of viral RNA-binding transcription factor activity | A process in which a host organism modulates the frequency, rate or extent of the activity of a viral RNA-binding transcription factor  **Exact synonym:** modulation by host of viral Tat activity |

*Keywords assigned to proteins because they are involved in a particular biological process.; GO version 2020-06-23; Annotation set created on 2020-06-15 08:11

ESCRT – the endosomal sorting complexes required for transport

CRISPR – Clustered Regularly Interspaced Short Palindromic Repeats
