# Supplementary material for: miRNA expression patterns in blood leukocytes and milk somatic cells of goats infected with small ruminant lentivirus (SRLV)
Source: Sci Rep. 2022 Aug 2;12:13239. doi: 10.1038/s41598-022-17276-y (PMC9344810; doi:10.1038/s41598-022-17276-y)
Supplement: Supplementary file 19 — Supplementary Table S14. [file 41598_2022_17276_MOESM19_ESM.docx]

**Table S14 a-c. Gene Ontology (GO) analysis of target genes for mir-141-3p throughout the miRNet software**

**Table S13a Biological processes**

| **No.** | **Name of biological process** | **Hits** | **Pval** | **adj.Pval** |
| --- | --- | --- | --- | --- |
| 1 | regulation of protein kinase activity | 16 | 0.000263 | 0.006741667 |
| 2 | positive regulation of cellular metabolic process | 37 | 0.000358 | 0.006741667 |
| 3 | regulation of kinase activity | 16 | 0.000525 | 0.006741667 |
| 4 | positive regulation of metabolic process | 38 | 0.000606 | 0.006741667 |
| 5 | positive regulation of nucleobase-containing compound metabolic process | 25 | 0.000623 | 0.006741667 |
| 6 | G1 phase of mitotic cell cycle | 4 | 0.000659 | 0.006741667 |
| 7 | embryo development | 20 | 0.000746 | 0.006741667 |
| 8 | regulation of growth | 13 | 0.000748 | 0.006741667 |
| 9 | regulation of transferase activity | 16 | 0.00075 | 0.006741667 |
| 10 | G1 phase | 4 | 0.000773 | 0.006741667 |
| 11 | positive regulation of transcription, DNA-dependent | 22 | 0.000809 | 0.006741667 |
| 12 | positive regulation of cell differentiation | 22 | 0.000809 | 0.006741667 |
| 13 | vasculature development | 13 | 0.00109 | 0.008384615 |
| 14 | stress-activated protein kinase signaling cascade | 14 | 0.00123 | 0.008785714 |
| 15 | regulation of protein phosphorylation | 8 | 0.00154 | 0.009611111 |
| 16 | positive regulation of RNA metabolic process | 18 | 0.0016 | 0.009611111 |
| 17 | regulation of JNK cascade | 22 | 0.00172 | 0.009611111 |
| 18 | growth | 6 | 0.00173 | 0.009611111 |
| 19 | cell proliferation | 16 | 0.00189 | 0.009947368 |
| 20 | negative regulation of transcription from RNA polymerase II promoter | 28 | 0.00216 | 0.0105 |
| 21 | regulation of cell differentiation | 12 | 0.0025 | 0.0105 |
| 22 | positive regulation of cell cycle | 21 | 0.00259 | 0.0105 |
| 23 | heart development | 5 | 0.00272 | 0.0105 |
| 24 | protein phosphorylation | 11 | 0.00282 | 0.0105 |
| 25 | tissue morphogenesis | 23 | 0.00304 | 0.0105 |
| 26 | positive regulation of transcription from RNA polymerase II promoter | 12 | 0.00306 | 0.0105 |
| 27 | glucose catabolic process | 15 | 0.00309 | 0.0105 |
| 28 | phosphorylation | 5 | 0.00316 | 0.0105 |
| 29 | angiogenesis | 25 | 0.0033 | 0.0105 |
| 30 | cell cycle arrest | 10 | 0.00333 | 0.0105 |
| 31 | regulation of phosphorylation | 10 | 0.00344 | 0.0105 |
| 32 | positive regulation of developmental process | 18 | 0.00373 | 0.0105 |
| 33 | tube development | 15 | 0.00376 | 0.0105 |
| 34 | interphase of mitotic cell cycle | 11 | 0.00377 | 0.0105 |
| 35 | negative regulation of transcription, DNA-dependent | 10 | 0.00386 | 0.0105 |
| 36 | regulation of protein modification process | 17 | 0.00392 | 0.0105 |
| 37 | anatomical structure formation involved in morphogenesis | 17 | 0.00392 | 0.0105 |
| 38 | interphase | 20 | 0.00399 | 0.0105 |
| 39 | enzyme linked receptor protein signaling pathway | 29 | 0.00424 | 0.01087179 |
| 40 | negative regulation of cell cycle | 10 | 0.00438 | 0.01095 |
| 41 | regulation of cellular protein metabolic process | 19 | 0.00457 | 0.011 |
| 42 | negative regulation of RNA metabolic process | 11 | 0.00462 | 0.011 |
| 43 | rhythmic process | 23 | 0.0055 | 0.0127907 |
| 44 | regulation of developmental process | 17 | 0.00566 | 0.01286364 |
| 45 | organ morphogenesis | 6 | 0.00709 | 0.01537255 |
| 46 | circadian rhythm | 26 | 0.0072 | 0.01537255 |
| 47 | JNK cascade | 16 | 0.00742 | 0.01537255 |
| 48 | regulation of MAPK cascade | 4 | 0.00743 | 0.01537255 |
| 49 | regulation of cell cycle | 6 | 0.00778 | 0.01537255 |
| 50 | regulation of transcription from RNA polymerase II promoter | 11 | 0.00783 | 0.01537255 |
| 51 | epithelial cell differentiation | 15 | 0.00784 | 0.01537255 |
| 52 | phosphatidylinositol-mediated signaling | 23 | 0.00801 | 0.01540385 |
| 53 | Ras protein signal transduction | 8 | 0.00818 | 0.01543396 |
| 54 | cellular response to stress | 5 | 0.00848 | 0.0157037 |
| 55 | anatomical structure morphogenesis | 7 | 0.00873 | 0.01587273 |
| 56 | tube morphogenesis | 23 | 0.00906 | 0.01612069 |
| 57 | regulation of protein metabolic process | 35 | 0.00927 | 0.01612069 |
| 58 | myeloid leukocyte differentiation | 8 | 0.00935 | 0.01612069 |
| 59 | transcription from RNA polymerase II promoter | 25 | 0.00965 | 0.01618333 |
| 60 | transcription initiation from RNA polymerase II promoter | 5 | 0.00971 | 0.01618333 |
| 61 | regulation of RNA metabolic process | 26 | 0.0101 | 0.01655738 |
| 62 | morphogenesis of an epithelium | 6 | 0.0108 | 0.01741935 |
| 63 | regulation of gene expression | 45 | 0.0111 | 0.01761905 |
| 64 | negative regulation of nucleobase-containing compound metabolic process | 9 | 0.0125 | 0.01953125 |
| 65 | ER-nucleus signaling pathway | 50 | 0.0129 | 0.01984615 |
| 66 | positive regulation of protein modification process | 17 | 0.0145 | 0.02116883 |
| 67 | transmembrane receptor protein tyrosine kinase signaling pathway | 4 | 0.0146 | 0.02116883 |
| 68 | intracellular protein kinase cascade | 14 | 0.0149 | 0.02116883 |
| 69 | intracellular steroid hormone receptor signaling pathway | 13 | 0.0151 | 0.02116883 |
| 70 | cellular carbohydrate catabolic process | 17 | 0.0151 | 0.02116883 |
| 71 | positive regulation of cell proliferation | 4 | 0.0155 | 0.02116883 |
| 72 | regulation of anatomical structure morphogenesis | 3 | 0.0156 | 0.02116883 |
| 73 | regulation of transcription, DNA-dependent | 13 | 0.0157 | 0.02116883 |
| 74 | protein targeting | 12 | 0.0158 | 0.02116883 |
| 75 | regulation of apoptotic process | 43 | 0.0163 | 0.02116883 |
| 76 | regulation of lipid metabolic process | 43 | 0.0163 | 0.02116883 |
| 77 | apoptotic process | 43 | 0.0163 | 0.02116883 |
| 78 | androgen receptor signaling pathway | 10 | 0.0173 | 0.02217949 |
| 79 | regulation of chromosome organization | 21 | 0.018 | 0.02268293 |
| 80 | carbohydrate catabolic process | 6 | 0.0183 | 0.02268293 |
| 81 | regulation of programmed cell death | 27 | 0.0186 | 0.02268293 |
| 82 | positive regulation of cellular component organization | 27 | 0.0186 | 0.02268293 |
| 83 | response to endogenous stimulus | 3 | 0.0192 | 0.02313253 |
| 84 | regulation of cell morphogenesis | 4 | 0.02 | 0.02314607 |
| 85 | programmed cell death | 5 | 0.0201 | 0.02314607 |
| 86 | positive regulation of multicellular organismal process | 21 | 0.0203 | 0.02314607 |
| 87 | DNA-dependent transcription, initiation | 10 | 0.0205 | 0.02314607 |
| 88 | mesoderm development | 19 | 0.0206 | 0.02314607 |
| 89 | cell morphogenesis involved in differentiation | 7 | 0.0206 | 0.02314607 |
| 90 | activation of protein kinase activity | 27 | 0.0216 | 0.02384615 |
| 91 | insulin receptor signaling pathway | 11 | 0.0217 | 0.02384615 |
| 92 | regulation of multicellular organismal process | 6 | 0.0221 | 0.02387097 |
| 93 | regulation of cellular metabolic process | 4 | 0.0222 | 0.02387097 |
| 94 | tissue development | 13 | 0.0228 | 0.02425532 |
| 95 | MAPK cascade | 6 | 0.0232 | 0.02442105 |

**Table S13b. Molecular functions**

| **No.** | **Name of molecular function** | **Hits** | **Pval** | **adj.Pval** |
| --- | --- | --- | --- | --- |
| 1 | transcription corepressor activity | 9 | 0.0000578 | 0.00578 |
| 2 | positive regulation of transcription, DNA-dependent | 22 | 0.000602 | 0.0275 |
| 3 | receptor signaling protein serine/threonine kinase activity | 4 | 0.000893 | 0.0275 |
| 4 | protein binding transcription factor activity | 13 | 0.0014 | 0.0275 |
| 5 | protein serine/threonine phosphatase activity | 4 | 0.00153 | 0.0275 |
| 6 | protein complex binding | 9 | 0.00204 | 0.0275 |
| 7 | transcription cofactor activity | 12 | 0.00208 | 0.0275 |
| 8 | receptor signaling protein activity | 5 | 0.0022 | 0.0275 |
| 9 | sequence-specific DNA binding | 14 | 0.00293 | 0.02783333 |
| 10 | negative regulation of transcription, DNA-dependent | 17 | 0.00314 | 0.02783333 |
| 11 | RNA polymerase II transcription cofactor activity | 4 | 0.00314 | 0.02783333 |
| 12 | transcription factor binding | 11 | 0.00334 | 0.02783333 |
| 13 | phosphoprotein phosphatase activity | 7 | 0.0042 | 0.03230769 |
| 14 | insulin-like growth factor receptor binding | 2 | 0.00581 | 0.0415 |
| 15 | protein domain specific binding | 11 | 0.00678 | 0.04505882 |
| 16 | MAP kinase activity | 2 | 0.00757 | 0.04505882 |
| 17 | transcription from RNA polymerase II promoter | 26 | 0.00766 | 0.04505882 |
| 18 | histone acetyltransferase activity | 3 | 0.00913 | 0.04615 |
| 19 | kinase activator activity | 3 | 0.00913 | 0.04615 |
| 20 | mRNA binding | 4 | 0.00923 | 0.04615 |

**Table S13c. Cellular componets**

| **No.** | **Name of cellular process** | **Hits** | **Pval** | **adj.Pval** |
| --- | --- | --- | --- | --- |
| 1 | nucleoplasm | 30 | 0.0000634 | 0.00327 |
| 2 | nuclear lumen | 39 | 0.0000654 | 0.00327 |
| 3 | membrane-enclosed lumen | 43 | 0.00074 | 0.02466667 |
| 4 | organelle lumen | 42 | 0.001 | 0.025 |
| 5 | nuclear part | 41 | 0.0014 | 0.028 |
| 6 | nucleoplasm part | 16 | 0.00208 | 0.03466667 |
| 7 | nucleus | 76 | 0.00284 | 0.039625 |
| 8 | PML body | 4 | 0.00317 | 0.039625 |
