# Supplementary material for: miRNA expression patterns in blood leukocytes and milk somatic cells of goats infected with small ruminant lentivirus (SRLV)
Source: Sci Rep. 2022 Aug 2;12:13239. doi: 10.1038/s41598-022-17276-y (PMC9344810; doi:10.1038/s41598-022-17276-y)
Supplement: Supplementary file 20 — Supplementary Table S15. [file 41598_2022_17276_MOESM20_ESM.docx]

**Table S15. Kyoto encyclopedia of genes and genomes (KEGG) pathway analysis for target genes of all miRNAs expressed in milk somatic cells (MSC)**

| **No.** | **Name** | **Hits** | **P-value** | **adj. P-value** |
| --- | --- | --- | --- | --- |
| 1 | Pathways in cancer | 61 | 3.6e-21 | 3.6e-19 |
| 2 | Focal adhesion | 44 | 3.59e-17 | 1.795e-15 |
| 3 | Prostate cancer | 26 | 8.02e-14 | 2.673333e-12 |
| 4 | Chronic myeloid leukemia | 22 | 6.07e-12 | 1.5175e-10 |
| 5 | Glioma | 20 | 3.81e-11 | 7.62e-10 |
| 6 | Small cell lung cancer | 21 | 3.4e-10 | 5.666667e-9 |
| 7 | Melanoma | 18 | 5.69e-9 | 8.128571e-8 |
| 8 | Colorectal cancer | 15 | 1.27e-8 | 1.5875e-7 |
| 9 | ECM-receptor interaction | 19 | 3.54e-8 | 3.933333e-7 |
| 10 | Neurotrophin signaling pathway | 23 | 5.7e-8 | 5.7e-7 |
| 11 | Toxoplasmosis | 19 | 2.01e-7 | 0.000001827273 |
| 12 | Cell cycle | 22 | 2.97e-7 | 0.000002369231 |
| 13 | Pancreatic cancer | 16 | 3.08e-7 | 0.000002369231 |
| **14** | **Epstein-Barr virus infection** | 18 | 7.1e-7 | 0.000005071429 |
| 15 | ErbB signaling pathway | 17 | 0.00000175 | 0.00001166667 |
| 16 | Acute myeloid leukemia | 13 | 0.00000501 | 0.0000313125 |
| 17 | Renal cell carcinoma | 13 | 0.00000918 | 0.000054 |
| **18** | **HTLV-I infection** | 26 | 0.0000106 | 0.00005578947 |
| 19 | Endometrial cancer | 11 | 0.0000106 | 0.00005578947 |
| 20 | MAPK signaling pathway | 31 | 0.0000145 | 0.0000725 |
| 21 | Chagas disease (American trypanosomiasis) | 15 | 0.0000463 | 0.0002204762 |
| 22 | Progesterone-mediated oocyte maturation | 14 | 0.0000541 | 0.0002459091 |
| 23 | Measles | 16 | 0.0000641 | 0.0002786957 |
| 24 | Fc epsilon RI signaling pathway | 13 | 0.000111 | 0.0004625 |
| **25** | **NOD-like receptor signaling pathway** | 10 | 0.000173 | 0.000692 |
| 26 | Insulin signaling pathway | 18 | 0.000239 | 0.0009192308 |
| 27 | Non-small cell lung cancer | 10 | 0.00029 | 0.001074074 |
| **28** | **Influenza A** | 15 | 0.000391 | 0.001396429 |
| 29 | mTOR signaling pathway | 9 | 0.00043 | 0.001482759 |
| **30** | **T cell receptor signaling pathway** | 14 | 0.000502 | 0.001673333 |
| **31** | **Jak-STAT signaling pathway** | 14 | 0.000558 | 0.0017875 |
| 32 | Bladder cancer | 7 | 0.000572 | 0.0017875 |
| 33 | p53 signaling pathway | 11 | 0.000695 | 0.002106061 |
| 34 | Protein processing in endoplasmic reticulum | 16 | 0.00101 | 0.002970588 |
| 35 | Axon guidance | 15 | 0.00112 | 0.003200000 |
| 36 | Osteoclast differentiation | 15 | 0.00122 | 0.003388889 |
| 37 | B cell receptor signaling pathway | 11 | 0.0016 | 0.004324324 |
| **38** | **Hepatitis C** | 13 | 0.00194 | 0.005105263 |
| 39 | Arrhythmogenic right ventricular cardiomyopathy (ARVC) | 4 | 0.002600 | 0.006666667 |
| 40 | Thyroid cancer | 6 | 0.00275 | 0.006875 |
| 41 | GnRH signaling pathway | 12 | 0.00334 | 0.008146341 |
| 42 | Prion diseases | 5 | 0.00385 | 0.009166667 |
| 43 | Bacterial invasion of epithelial cells | 8 | 0.00805 | 0.01872093 |
| 44 | Tuberculosis | 17 | 0.00896 | 0.02036364 |
| 45 | TGF-beta signaling pathway | 10 | 0.0116 | 0.02565217 |
| **46** | **Toll-like receptor signaling pathway** | 11 | 0.0118 | 0.02565217 |
| 47 | Carbohydrate digestion and absorption | 4 | 0.0125 | 0.02659574 |
| 48 | Regulation of actin cytoskeleton | 17 | 0.0137 | 0.02854167 |
| 49 | Amyotrophic lateral sclerosis (ALS) | 6 | 0.0147 | 0.03000000 |
| 50 | VEGF signaling pathway | 9 | 0.0168 | 0.03333333 |
| 51 | Gap junction | 10 | 0.017 | 0.03333333 |
| **52** | **Apoptosis** | 9 | 0.0283 | 0.05442308 |
