# Supplementary material for: miRNA expression patterns in blood leukocytes and milk somatic cells of goats infected with small ruminant lentivirus (SRLV)
Source: Sci Rep. 2022 Aug 2;12:13239. doi: 10.1038/s41598-022-17276-y (PMC9344810; doi:10.1038/s41598-022-17276-y)
Supplement: Supplementary file 21 — Supplementary Table S16. [file 41598_2022_17276_MOESM21_ESM.docx]

**Table S16. Kyoto encyclopedia of genes and genomes (KEGG) pathway analysis for target genes of all miRNAs expressed in blood leukocytes (BL)**

| **No.** | **Name** | **Hits** | **Pval** | **adj.Pval** |
| --- | --- | --- | --- | --- |
| 1 | Pathways in cancer | 88 | 5.26e-16 | 5.26e-14 |
| 2 | Prostate cancer | 36 | 2.75e-12 | 1.375e-10 |
| 3 | Focal adhesion | 58 | 3.3e-11 | 1.1e-9 |
| **4** | **HTLV-I infection** | 56 | 2.69e-10 | 6.725e-9 |
| 5 | Chronic myeloid leukemia | 29 | 1.24e-9 | 2.48e-8 |
| 6 | Small cell lung cancer | 30 | 3.28e-9 | 5.466667e-8 |
| 7 | Glioma | 25 | 3.83e-8 | 5.471429e-7 |
| 8 | Colorectal cancer | 21 | 5.21e-8 | 6.5125e-7 |
| 9 | Pancreatic cancer | 25 | 1.52e-7 | 0.000001688889 |
| 10 | Toxoplasmosis | 30 | 1.75e-7 | 0.00000175 |
| 11 | Neurotrophin signaling pathway | 35 | 5.28e-7 | 0.0000048 |
| 12 | Cell cycle | 35 | 6.54e-7 | 0.0000052 |
| 13 | Renal cell carcinoma | 22 | 6.76e-7 | 0.0000052 |
| 14 | Epstein-Barr virus infection | 28 | 0.00000134 | 0.000009571429 |
| **15** | **Influenza A** | 31 | 0.00000156 | 0.0000104 |
| 16 | p53 signaling pathway | 22 | 0.00000748 | 0.000044 |
| 17 | Melanoma | 22 | 0.00000748 | 0.000044 |
| 18 | Bladder cancer | 13 | 0.0000107 | 0.00005684211 |
| **19** | **MAPK signaling pathway** | 56 | 0.0000108 | 0.00005684211 |
| 20 | Osteoclast differentiation | 31 | 0.0000175 | 0.0000852381 |
| 21 | Non-small cell lung cancer | 18 | 0.0000179 | 0.0000852381 |
| 22 | Measles | 27 | 0.0000451 | 0.000205 |
| **23** | **Apoptosis** | 23 | 0.0000766 | 0.0003330435 |
| 24 | Chagas disease (American trypanosomiasis) | 24 | 0.0000864 | 0.00036 |
| 25 | Endometrial cancer | 15 | 0.00011 | 0.00044 |
| 26 | ErbB signaling pathway | 23 | 0.000168 | 0.0006461538 |
| **27** | **Hepatitis C** | 25 | 0.000232 | 0.0008592593 |
| 28 | TGF-beta signaling pathway | 22 | 0.000268 | 0.0009241379 |
| 29 | ECM-receptor interaction | 22 | 0.000268 | 0.0009241379 |
| 30 | Insulin signaling pathway | 31 | 0.000305 | 0.001016667 |
| 31 | Axon guidance | 27 | 0.000614 | 0.001980645 |
| 32 | Regulation of actin cytoskeleton | 37 | 0.000778 | 0.00243125 |
| 33 | Progesterone-mediated oocyte maturation | 20 | 0.000961 | 0.002912121 |
| **34** | **T cell receptor signaling pathway** | 23 | 0.00106 | 0.003085714 |
| **35** | **B cell receptor signaling pathway** | 19 | 0.00108 | 0.003085714 |
| **36** | **Jak-STAT signaling pathway** | 23 | 0.00123 | 0.003416667 |
| 37 | Toll-like receptor signaling pathway | 22 | 0.00216 | 0.005837838 |
| 38 | Acute myeloid leukemia | 15 | 0.00236 | 0.006210526 |
| 39 | Protein processing in endoplasmic reticulum | 27 | 0.00252 | 0.006461538 |
| **40** | **NOD-like receptor signaling pathway** | 13 | 0.0042 | 0.0105 |
| **41** | **Herpes simplex infection** | 22 | 0.00475 | 0.01158537 |
| 42 | Bacterial invasion of epithelial cells | 14 | 0.0054 | 0.01285714 |
| **43** | **mTOR signaling pathway** | 12 | 0.0056 | 0.01302326 |
| 44 | Prion diseases | 7 | 0.00906 | 0.02028889 |
| **45** | **Viral myocarditis** | 8 | 0.00913 | 0.02028889 |
| 46 | RIG-I-like receptor signaling pathway | 12 | 0.0115 | 0.02479167 |
| 47 | Wnt signaling pathway | 27 | 0.0118 | 0.02479167 |
| 48 | Circadian rhythm - mammal | 7 | 0.0119 | 0.02479167 |
| 49 | Thyroid cancer | 8 | 0.0146 | 0.0294 |
| 50 | Amyotrophic lateral sclerosis (ALS) | 10 | 0.0147 | 0.0294 |
| 51 | GnRH signaling pathway | 19 | 0.0151 | 0.02960784 |
| 52 | Carbohydrate digestion and absorption | 6 | 0.0155 | 0.02980769 |
| 53 | Leishmaniasis | 12 | 0.0158 | 0.02981132 |
| **54** | **Chemokine signaling pathway** | 33 | 0.0165 | 0.03055556 |
| 55 | Pertussis | 12 | 0.0183 | 0.03327273 |
| 56 | Fc gamma R-mediated phagocytosis | 19 | 0.0207 | 0.03696429 |
| 57 | Shigellosis | 11 | 0.0212 | 0.03719298 |
| 58 | Salmonella infection | 15 | 0.0224 | 0.03862069 |
| 59 | Type II diabetes mellitus | 11 | 0.0246 | 0.04169492 |
| 60 | Epithelial cell signaling in Helicobacter pylori infection | 9 | 0.0281 | 0.04683333 |
